# Distinct antibody repertoires against endemic human coronaviruses in children and adults

**DOI:** 10.1172/jci.insight.144499

**Published:** 2021-02-22

**Authors:** Taushif Khan, Mahbuba Rahman, Fatima Al Ali, Susie S. Y. Huang, Manar Ata, Qian Zhang, Paul Bastard, Zhiyong Liu, Emmanuelle Jouanguy, Vivien Béziat, Aurélie Cobat, Gheyath K. Nasrallah, Hadi M. Yassine, Maria K. Smatti, Amira Saeed, Isabelle Vandernoot, Jean-Christophe Goffard, Guillaume Smits, Isabelle Migeotte, Filomeen Haerynck, Isabelle Meyts, Laurent Abel, Jean-Laurent Casanova, Mohammad R. Hasan, Nico Marr

**Affiliations:** 1 Research Branch, Sidra Medicine, Doha, Qatar.; 2 St. Giles Laboratory of Human Genetics of Infectious Diseases, Rockefeller Branch, Rockefeller University, New York, New York, USA.; 3 Laboratory of Human Genetics of Infectious Diseases, Necker Branch, INSERM U1163, Necker Hospital for Sick Children, Paris, France.; 4 University of Paris, Imagine Institute, Paris, France.; 5 College of Health Sciences, QU Health, Qatar University, Doha, Qatar.; 6 Biomedical Research Center, Qatar University, Doha, Qatar.; 7 Department of Pathology, Sidra Medicine, Doha, Qatar.; 8 Center of Human Genetics,; 9 Department of Internal Medicine, and; 10 Fonds de la Recherche Scientifique (FNRS) and Center of Human Genetics, Hôpital Erasme, Université Libre de Bruxelles, Brussels, Belgium.; 11 Department of Pediatric Pulmonology and Immunology, Department of Pediatrics and Internal Medicine, Center for Primary Immunodeficiencies Ghent, Jeffrey Modell Foundation Diagnostic and Research Center, Ghent University Hospital, Belgium.; 12 Laboratory for Inborn Errors of Immunity, Department of Microbiology, Immunology and Transplantation, and Department of Pediatrics, University Hospitals Leuven, KU Leuven, Belgium.; 13 Department of Pediatrics, University Hospitals Leuven, KU Leuven, Belgium.; 14 Howard Hughes Medical Institute, New York, New York, USA.; 15 Weill Cornell Medical College in Qatar, Doha, Qatar.; 16 College of Health and Life Sciences, Hamad Bin Khalifa University, Doha, Qatar.

**Keywords:** Immunology, Infectious disease, Immunoglobulins

## Abstract

Four endemic human coronaviruses (HCoVs) are commonly associated with acute respiratory infection in humans. B cell responses to these “common cold” viruses remain incompletely understood. Here we report a comprehensive analysis of CoV-specific antibody repertoires in 231 children and 1168 adults using phage immunoprecipitation sequencing. Seroprevalence of antibodies against endemic HCoVs ranged between approximately 4% and 27% depending on the species and cohort. We identified at least 136 novel linear B cell epitopes. Antibody repertoires against endemic HCoVs were qualitatively different between children and adults in that anti-HCoV IgG specificities more frequently found among children targeted functionally important and structurally conserved regions of the spike, nucleocapsid, and matrix proteins. Moreover, antibody specificities targeting the highly conserved fusion peptide region and S2′ cleavage site of the spike protein were broadly cross-reactive with peptides of epidemic human and nonhuman coronaviruses. In contrast, an acidic tandem repeat in the N-terminal region of the Nsp3 subdomain of the HCoV-HKU1 polyprotein was the predominant target of antibody responses in adult donors. Our findings shed light on the dominant species-specific and pan-CoV target sites of human antibody responses to coronavirus infection, thereby providing important insights for the development of prophylactic or therapeutic monoclonal antibodies and vaccine design.

## Introduction

Four endemic human-tropic coronaviruses (HCoVs) are commonly associated with respiratory illness in humans, namely HCoV-229E, -NL63, -OC43, and -HKU1 (1–4). Clinical outcomes of acute infection with these HCoVs range from mild upper respiratory tract infections in most patients, to viral bronchiolitis and pneumonia more rarely in patients, the latter requiring hospitalization (5). The ratio of more severe versus mild outcomes of acute infection with endemic HCoVs is largely comparable to that of other “common cold” viruses, such as human respiratory syncytial virus (HRSV), human rhinoviruses (HRVs), human adenoviruses, and human parainfluenza viruses, albeit with differences in seasonality and prevalence of the viruses depending on the species (5–7). In addition to the 4 endemic HCoVs, 3 human-tropic epidemic coronaviruses (CoVs) have emerged over the last 2 decades, namely severe acute respiratory syndrome–CoV (SARS-CoV) (8), Middle East respiratory syndrome–CoV (MERS-CoV) (9), and SARS-CoV-2 (10), the etiological agent of coronavirus disease 2019 (COVID-19), which has now reached pandemic proportions (11). Similar to endemic HCoVs, infection of humans with epidemic CoVs is associated with a wide range of outcomes but leads more frequently to severe clinical manifestations, such as acute respiratory distress syndrome (ARDS) (12–14). Phylogenetic analyses suggest that, similar to these epidemic CoVs, all endemic HCoVs are of zoonotic origin, and their possible ancestors share similar natural animal reservoirs and intermediate hosts (6). HCoV-229E may have been transferred from dromedary camels, similar to MERS-CoV, while HCoV-OC43 is thought to have emerged more recently from ancestors in domestic animals such as cattle or swine in the context of a pandemic at the end of the 19th century (6, 15).

The wide variability in transmissibility and clinical manifestations of infections by endemic and epidemic CoVs among humans remains poorly understood. On the population level, the case fatality rate is highest for MERS (approximately 34%– 37%), and several risk factors are associated with progression to ARDS in MERS, SARS, and COVID-19 cases, including old age (i.e., people aged 65 years or over), diabetes mellitus, hypertension, cancer, renal and lung disease, and coinfections (12, 16). Nonetheless, even MERS-CoV infection among humans can run a completely asymptomatic course in some cases, particularly among children (17–19). There is evidence that children are generally less susceptible to infection with epidemic CoVs, and once infected, they are less likely to experience severe outcomes compared with adults, although this important association and the underlying reasons remain poorly understood (12, 18, 20, 21). Importantly, it remains unclear to what extent preexisting immunity from past infections with endemic HCoVs provides some degree of cross-protection and affects clinical outcomes of infection with the epidemic SARS-CoV-2 or MERS-CoV. Our overall understanding of the immunity induced by natural infection with endemic HCoVs remains very limited. Serological studies have shown some degree of cross-reactive antibodies in patients with past CoV infections, but many of these studies were limited in sample size and often focused on specific viral antigens only (22–25). Depending on their binding affinity and specificities, such cross-reactive antibodies could have no effect on clinical outcomes, may provide protection from severe disease to some degree, or may lead to antibody-dependent enhancement of disease — the latter can be a major obstacle in vaccine development (26). Interestingly, 2 recent studies from independent groups have shown that a considerable proportion of individuals without a history of SARS-CoV-2 infection have SARS-CoV-2–reactive T cells, which suggests that cross-reactive T cell subsets originating from past infections by endemic HCoVs may play a role in the clinical course of infection with the phylogenetically related epidemic CoVs (27, 28). A systematic assessment to elucidate the immunodominant B cell antigen determinants of endemic HCoVs has not been done. We hypothesized that a fraction of the general population also have antibodies generated during past encounters with “common cold” coronaviruses that cross-react with proteins of epidemic CoVs. This may affect the dynamics of sporadic MERS outbreaks that mostly occur in the Middle East and the current COVID-19 pandemic.

## Results

To gain a deeper insight into human antibody responses to endemic HCoVs, we performed phage immunoprecipitation sequencing (PhIP-Seq) (29, 30) on previously collected serum or plasma samples obtained from 1431 human subjects from 3 cohorts. These included (a) healthy male adult blood donors (ABD) with diverse ethnic backgrounds and nationalities (Supplemental Figure 1A; supplemental material available online with this article; https://doi.org/10.1172/jci.insight.144499DS1); (b) adult male and female participants of a national cohort study — the Qatar Biobank (QBB) (31) — representing the general population (Supplemental Figure 1B); and (c) pediatric outpatients and inpatients who were tested for metabolic conditions unrelated to infection, chronic disease, or cancer (Methods and Supplemental Figure 1C). The samples were collected prior to the current COVID-19 outbreak (Methods). In brief, PhIP-Seq allowed us to obtain comprehensive antiviral antibody repertoires across individuals in our 3 human cohorts using phage display of oligonucleotide-encoded peptidomes, followed by immunoprecipitation and massive parallel sequencing (29, 30). The VirScan phage library used for PhIP-Seq in the present study comprised peptides derived from viral proteins — each represented by peptide tiles of up to 56 amino acids in length that overlap by 28 amino acids — which collectively encompass the proteomes of a large number of viral species, including HCoV-229E, -NL63, -HKU1, and -OC43 (29, 30). Proteins of endemic HCoVs that were represented in the VirScan phage library included the ORF1ab replicase polyprotein (pp1ab), the spike glycoprotein (S), the matrix glycoprotein (M), the nucleocapsid protein (N), and gene products of the species- and strain-specific open reading frames (ORFs) encoded in the 3′ region of the viral genomes (Supplemental Table 1). Of note, we utilized an expanded version of the VirScan phage library (32, 33), which also encompassed peptides from a number of proteins of human epidemic and nonhuman CoV isolates, including MERS-CoV, SARS-CoV, as well as bat, bovine, porcine, and feline isolates belonging to the alpha- and beta-CoV genera, albeit with varying coverage of the viral peptidomes owing to the limitation in available sequence data for the latter isolates in UniProt (Supplemental Table 1). SARS-CoV-2 peptides were not included in the VirScan phage library used in our study.

We were able to obtain antibody repertoires for 1399 individuals from the human cohorts described above (Supplemental Table 2). Using stringent filter criteria (Methods), we identified a total of 417 out of 2498 peptides and potential antigens from endemic HCoVs with our screen that were significantly enriched in at least 3 of all 1399 analyzed individuals. A total of 103 peptides from endemic HCoVs were enriched in at least 1% of the samples and therefore considered to contain potentially immunodominant regions (Supplemental Table 3). Only 33 of the 417 peptides enriched in at least 3 samples shared linear sequence homology with epitopes that have previously been reported (34) (Supplemental Figure 2). To estimate number of newly identified linear B cell epitopes, we assigned each CoV-derived peptide to clusters of peptides that share at least 7 amino acids linear sequence identity — the estimated size of a linear B cell epitope (Methods). The enriched peptides could be assigned to 149 clusters for which at least 2 peptides shared linear sequence identity of at least 7 amino acids (Supplemental Tables 3 and 4). Only 13 clusters also shared at least 7 amino acids linear sequence identity with known linear B cell epitopes. Consequently, we have identified a minimum of 136 new linear epitopes, including 25 new immunodominant linear B cell epitopes — i.e., B cell epitopes targeted in at least 1% of all individuals and not already reported in the Immune Epitope Database: https://www.iedb.org (34) (Supplemental Table 3).

**Figure 1:**
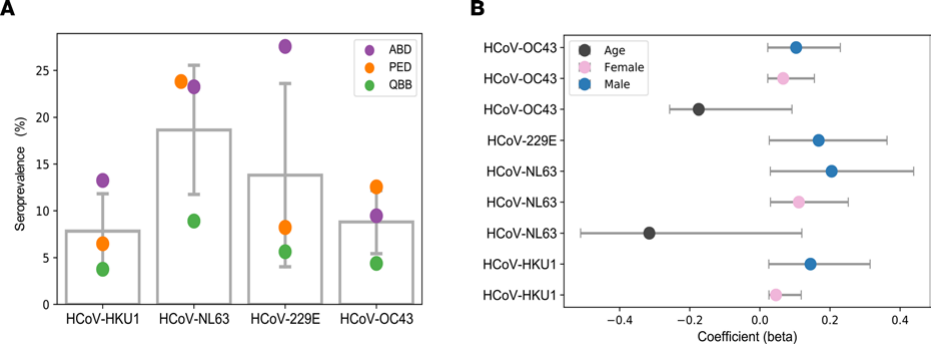
Seroprevalence of endemic HCoVs. (**A**) Dot plot depicting the seroprevalence of the 4 endemic HCoVs among subjects included in the downstream analysis (*n* = 1399) after stratification by cohort. Gray bars depict the mean seroprevalence value for each species; error bars depict the SD. QBB, Qatar Biobank cohort; ABD, adult (male) blood bank donors; PED, pediatric study subjects. (**B**) Coefficient of association (β) with 95% CI of seroprevalence for each HCoV with male sex (blue), female sex (pink), or age (black). Only features that had a *P* value of association less than or equal to 0.001 are shown.

**Figure 2:**
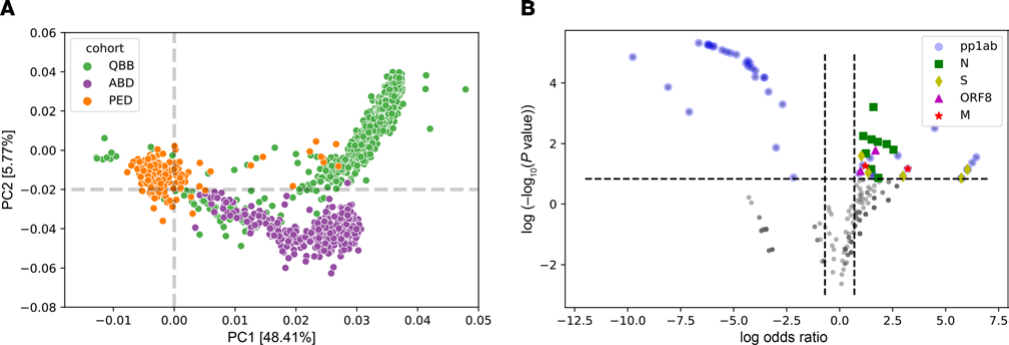
Qualitative differences in antibody repertoires among cohorts and age groups. (**A**) Principal component analysis of 417 peptides from endemic HCoVs that were found to be enriched in at least 3 samples. QBB (*n* = 798); ABD (*n* = 370); PED (*n* = 231). (**B**) Differential enrichment analysis to determine the peptides that are either more or less frequently enriched in children versus adults (including subjects of both adult cohorts, namely QBB and ABD). We considered a peptide significantly more or less frequently enriched among children if the OR was ≥ 2 or ≤ –2, respectively, and the *P* value was less than or equal to 0.005 (Fisher’s exact test). pp1ab, ORF1ab replicase polyprotein; S, spike glycoprotein; M, matrix glycoprotein; N, nucleocapsid protein; ORF8, open reading frame 8 protein.

Next we assessed the seroprevalence of HCoV-229E, -NL63, -HKU1, and -OC43 in the 3 cohorts separately. To do so, we computed species score values as described earlier (30, 32, 35) by counting the significantly enriched peptides for a given HCoV species that shared less than 7 amino acids linear sequence identity. We considered an individual seropositive for any of the endemic HCoVs if the number of nonhomologous peptides enriched in a given sample met our previously established species-specific cutoff value (Methods). Seroprevalence for endemic HCoVs ranged from approximately 4% to approximately 27%, depending on the species and cohort (Figure 1A), and also varied when stratifying the subjects by age group or sex (Supplemental Table 5). Interestingly, we found a marginal but significant negative association between age and seroprevalence of HCoV-OC43 (β = –0.175) and -NL63 (β = –0.315), as well as a marginally positive association between male sex and seroprevalence for any of the endemic HCoVs (β ≤ 0.2) (Figure 1B). The species score values (i.e., the antibody repertoire breadth for each HCoV species) did not differ substantially between seropositive individuals of our 3 cohorts (Supplemental Figure 3). However, principal component analysis revealed considerable qualitative differences in the antibody repertoires between our cohorts and in particular between pediatric and adult subjects (Figure 2A). For comparison, we also performed the same analysis on enriched peptides from other common respiratory viruses, including HRSV, HRV A, HRV B, and influenza B virus. As expected, seroprevalence was considerably higher (68% to 99%) for HRSV, HRV A, and HRV B and somewhat higher (29% to 47%) for influenza B virus (Supplemental Figure 4A). However, contrary to antiviral antibody responses to endemic HCoVs, we did not find considerable variance in the antibody repertoires to other respiratory viruses when comparing age groups and cohorts (Supplemental Figure 4B). We also analyzed the enriched antigenic peptides for each endemic HCoV species separately and found that most variance in the antibody repertoires between cohorts and age groups was attributable to past infections with HCoV-HKU1 and -229E (Supplemental Figure 4C). To determine the antibody specificities responsible for most of the variance in the antiviral response to endemic HCoVs between adults and children (i.e., to identify those peptides that were significantly more or less frequently enriched when comparing adult and pediatric donors), we applied Fisher’s exact test and computed log odds ratios (lod) for each of the significantly enriched peptides. We found that antibody specificities in samples of pediatric study subjects predominantly targeted different antigenic regions in the S protein (mean lod = 3.35, SD = 2.12) and the N protein (mean lod = 2.21, SD = 1.41) and diverse antigenic sites in pp1ab, whereas peptides encoding a single linear B cell epitope of pp1ab (cluster 22) appeared to be the predominant target of IgG antibodies among adult donors (mean lod = –4.7, SD = 1.16) (Figure 2B, Table 1, and Supplemental Table 6).

**Figure 3:**
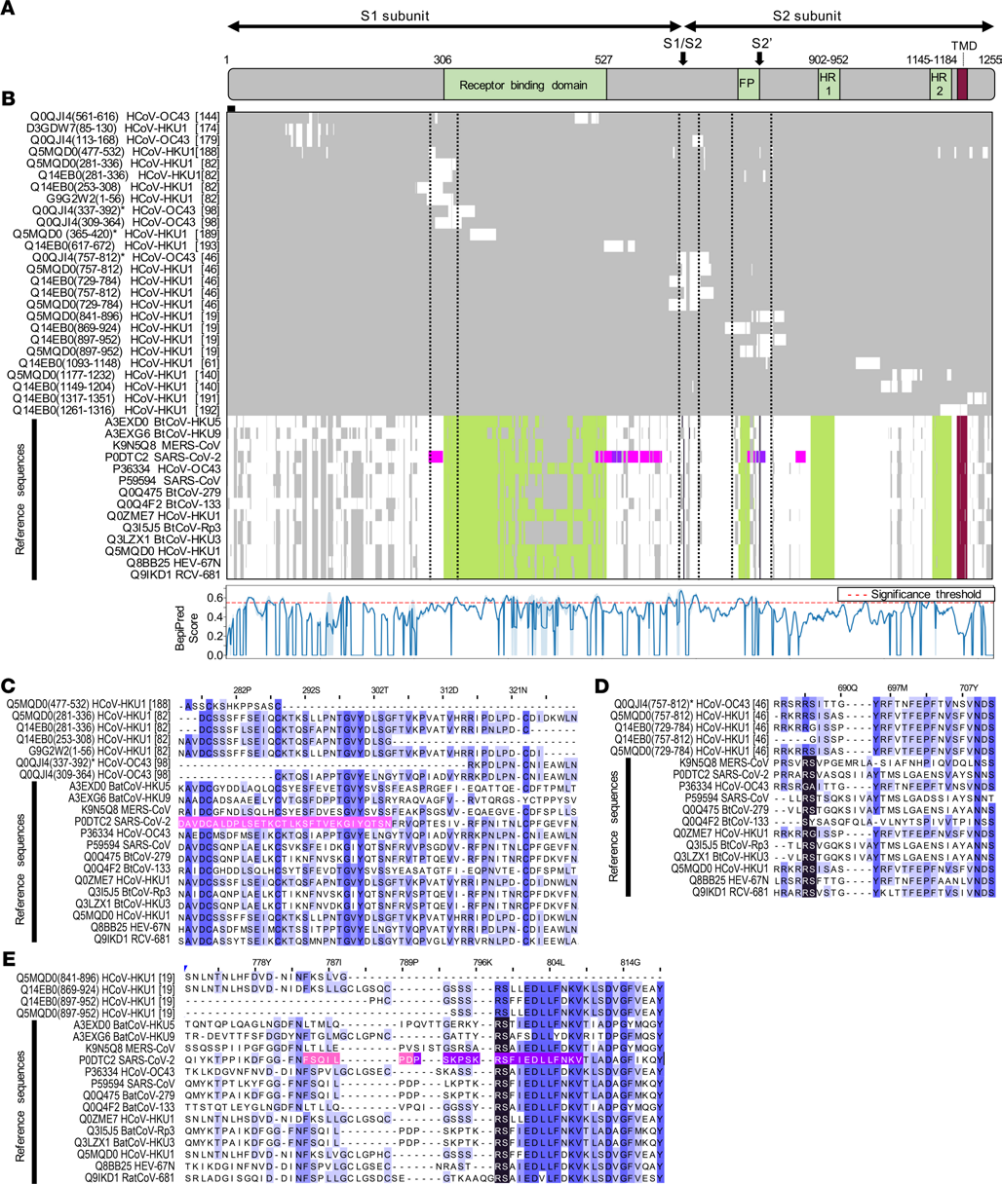
Antigenic regions and predicted antibody binding sites of the S protein. (**A**) Schematic representation of the S protein of SARS-CoV (UniProtKB entry P59594). Proteolytic cleavage sites are marked with arrows. FP, fusion peptide; HR, heptad repeats; TMD, transmembrane domain. (**B**) Overview of a multiple sequence alignment of immunodominant peptides with the full-length protein sequences of various alpha- and beta-CoVs (top). Row labels indicate the UniProtKB sequence identifier, start and end positions of enriched peptides (in parentheses), names of the organisms, and cluster numbers (in square brackets). Peptides for which differential enrichment between children and adults were statistically significant (*P* ≤ 0.005, Fisher’s exact test) and ORs were at least 2 are indicated with an asterisk. Colors indicate protein domains as shown in **A**, as well as predicted (pink) (42) and experimentally validated (purple) (56) linear SARS-CoV-2 B cell epitopes. Vertical dashed lines indicate boundaries of regions shown in **C**–**E**. The line plot (bottom) shows the mean BepiPred score (blue line) and SD (shaded) for the prediction of linear B cell epitopes among endemic HCoVs. The significance threshold of 0.55 has been marked with a dashed red line. (**C**–**E**) Selected regions of the multiple sequence alignment encompassing the N-terminal region of the receptor binding domain (**C**), the S1/S2 cleavage site (**D**), and the S2′ cleavage site (**E**). Amino acid positions on top are shown for UniProtKB entry P59594. Amino acids are marked in color to indicate the level of sequence identity (blue), the proteolytic cleavage sites (black), and linear SARS-CoV-2 B cell epitopes as shown in **B**. The full sequence alignment is shown in Supplemental Figure 5A.

**Figure 4:**
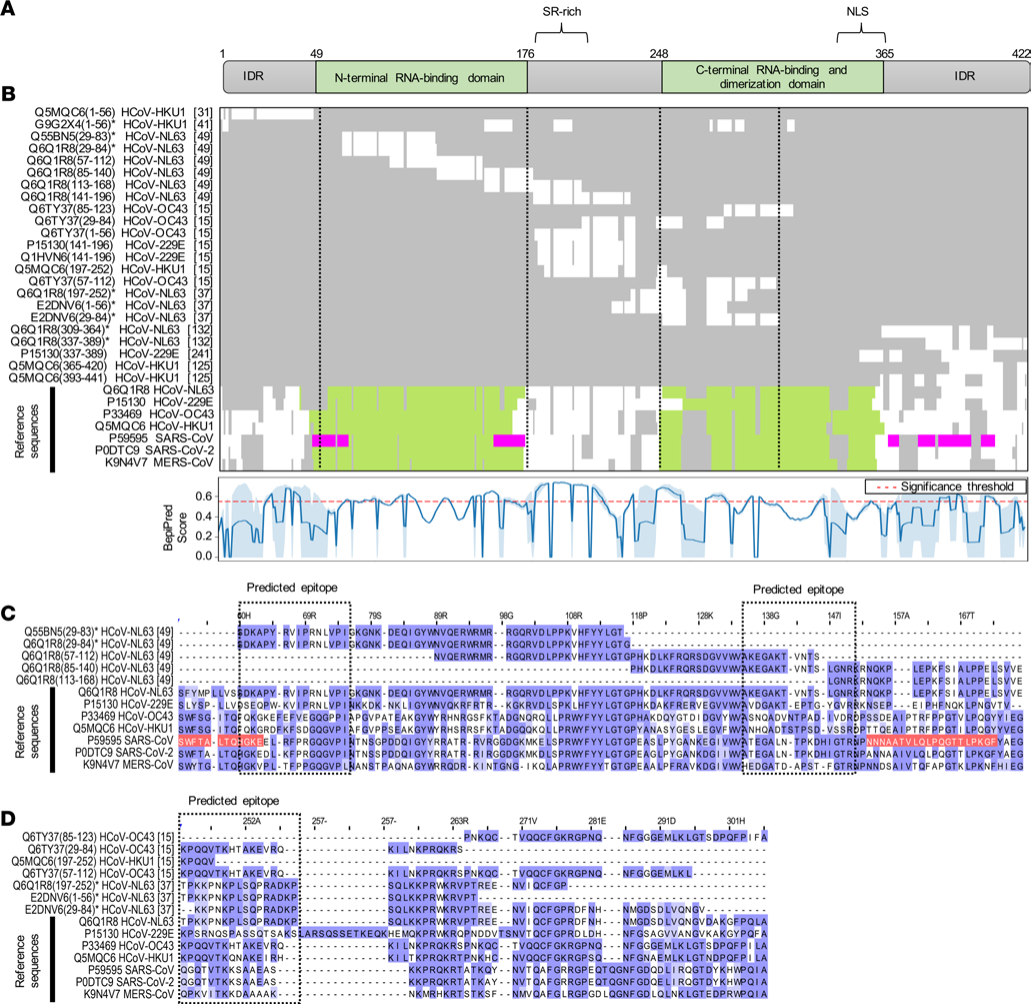
Antigenic regions and predicted antibody binding sites of the N protein. (**A**) Schematic representations of the N protein of SARS-CoV (UniProtKB entry P59595). SR-rich, serine- and arginine-rich motif; NLS, predicted nuclear localization sequence; IDR, intrinsically disordered region. (**B**) Overview of a multiple sequence alignment of immunodominant peptides with the full-length protein sequences of various alpha- and beta-CoVs (top). Row labels indicate the UniProtKB sequence identifier, start and end positions of enriched peptides (in parentheses), names of the organisms, and cluster numbers (in square brackets). Peptides for which differential enrichment between children and adults was statistically significant (*P* ≤ 0.005, Fisher’s exact test) and ORs at least ≥ 2 are indicated with an asterisk. Colors indicate protein domains as shown in **A** and predicted linear SARS-CoV-2 B cell epitopes (pink) (42). Vertical dashed lines indicate boundaries of the regions shown in **C** and **D**. The line plot (bottom) shows the mean BepiPred score (blue line) and SD (shaded) for the prediction of linear B cell epitopes among endemic HCoVs. The significance threshold of 0.55 has been marked with a dashed red line. (**C** and **D**) Selected regions of the multiple sequence alignment encompassing the N-terminal RNA binding domain (**C**) and C-terminal self-assembly domain (**D**). Amino acid positions on top are shown for UniProtKB reference sequence entry P59595. Amino acids are marked in color to indicate the level of sequence identity (blue) and linear SARS-CoV-2 B cell epitopes (red). The full multiple sequence alignment is shown in Supplemental Figure 5B.

Intriguingly, multiple sequence alignments of frequently enriched peptides with the full-length proteins of various CoVs revealed that antibody specificities predominantly found in pediatric study subjects targeted immunodominant epitopes that encode functionally important and highly conserved regions of the structural proteins. These included regions in the S1 subunit of the S protein that are important for receptor binding (36–39), as well as the regions resembling the proteolytic cleavage sites and fusion peptide of the S2 subunit (Figure 3 and Supplemental Figure 6). Of note, the immunodominant region spanning the furin-like S2′ cleavage site in the S2 subunit resembled one of the most conserved regions of the S protein, both in amino acid sequence (R¯SA[I/L]ED[I/L]LF) (Figure 3E) and in protein structure because it formed an accessible α-helix within the fusion peptide region (Supplemental Figure 6) (40). Moreover, we identified potential antibody binding sites in the N-terminal RNA binding domain, serine/arginine-rich region, and C-terminal dimerization domain of the N protein (Figure 4, A and B). Although the predicted antibody binding sites in the N-terminal RNA binding domain and the C-terminal dimerization domain of the N protein appeared to be less conserved between different species in the primary amino acid sequence (Figure 4, C and D), both domains were structurally conserved in the regions that we found to be immunodominant (Supplemental Figure 7). We also found that antibodies in children more frequently targeted the C-terminal domain of the M protein (Supplemental Figure 5C and Table 1) and the small accessory ORF8 protein (also known as N2) of HCoV-HKU1 (Table 1). Although ORF8 and N share the same coding sequence in the viral RNA genome, the reading frame is different and the amino acid sequences are not homologous. On the contrary, antibody specificities predominantly found in adults primarily targeted a region of the pp1ab that is specific to HCoV-HKU1 and contains an acidic tandem repeat (NDDE[D/H]VVTGD), which is located upstream of the papain-like protease 1 domain (Supplemental Figure 5D and Supplemental Table 6).

**Figure 5:**
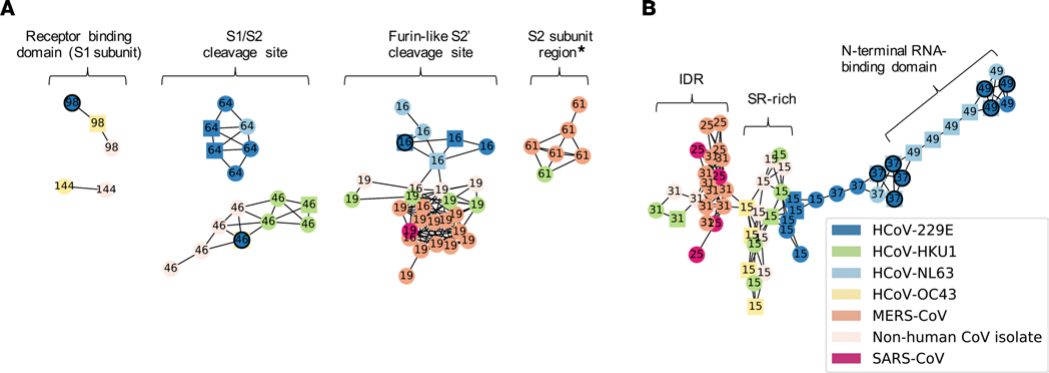
Network representation of enriched peptides from structural proteins targeted by cross-reactive antibodies. (**A**) Network representation of enriched S protein–derived peptides. (**B**) Network representation of enriched N protein–derived peptides. Each node represents an enriched peptide and the color indicates the species. Edges indicate at least 7 amino acids linear sequence identity between 2 nodes (i.e., peptides), the estimated size of a linear B cell epitope. Only networks of peptides derived from at least 2 species are shown. Labels indicate the cluster number to which each peptide has been assigned. Nodes are represented as spheres if the peptide had been frequently enriched. Nodes marked with a black circle indicate peptides for which differential enrichment between children and adults was statistically significant (*P* value ≤ 0.005, Fisher’s exact test) and ORs were at least 2. SR-rich, serine- and arginine-rich motif; IDR, intrinsically disordered region; asterisk, region between heptad repeat 1 and heptad repeat 2 of the S2 subunit.

Given the high degree of sequence conservation among some of the immunodominant regions in proteins of endemic HCoVs we have identified, we also explored the extent to which antibody specificities to these regions cross-react with peptides from epidemic CoVs and nonhuman CoV isolates. For this purpose, we assessed the enrichment of peptides derived from SARS-CoV, MERS-CoV, as well as bovine, porcine, bat, and feline CoV isolates (Supplemental Table 1), applying the same approach and stringent filter criteria as described above for peptides of endemic HCoVs. Indeed, we identified several S protein– and N protein–derived peptides from epidemic CoVs or nonhuman isolates that were significantly enriched in our PhIP-Seq assay and that shared sequence similarity with peptides from HCoVs (Figure 5). As expected based on the results from multiple sequence alignments described above, antibody specificities targeting the highly conserved amino acid motif (RSA[I/L]ED[I/L]LF) spanning the furin-like S2′ cleavage site of the S protein were broadly cross-reactive to several orthologous peptides from MERS-CoV, SARS-CoV, and nonhuman CoV isolates (Figure 5A). Cross-reactivity of antibodies targeting other functionally important but less conserved regions of the S protein also appeared more restricted (Figure 5A). Antibody specificities targeting the N protein also showed considerable cross-reactivity with peptides from MERS-CoV, SARS-CoV, and nonhuman CoV isolates. However, the latter cross-reactive antibodies mainly targeted regions rich in serine and arginine residues, with low-complexity sequences and very limited structural conservation, particularly an IDR at the N terminus of the N protein (Figure 5B and Supplemental Figure 9) that lacks a tertiary structure (41). We also detected cross-reactive antibodies targeting the serine- and arginine-rich linker region of the N protein; however, cross-reactivity to this region was largely restricted to peptides derived from nonhuman CoV isolates of domestic animals (Figure 5B), which are more closely related to HCoV-OC43 (6).

Finally, we assessed plasma samples of a previously healthy 49-year-old female adult patient who suffered from severe ARDS from SARS-CoV-2 infection, requiring prolonged hospitalization and intensive care. A first sample was obtained 25 days after onset of symptoms and 18 days after intensive care unit admission. A second sample was obtained 1 month later, 1 week after discharge. We compared the antibody profiles at both time points with samples obtained from uninfected family members of the patient, as well as an age- and sex-matched unrelated control. In agreement with what we found in some subjects with a history of endemic HCoV infection, the patient with severe COVID-19 had detectable antibodies that cross-reacted with peptides from SARS-CoV and nonhuman CoVs encoding the furin-like S2′ cleavage site and heptad repeat 2 region of the S protein, peptides from the C-terminal region of the HCoV-HKU1 N protein downstream of the dimerization domain, as well as 2 antigenic sites of the MERS-CoV pp1a (Supplemental Figure 8). We confirmed these findings by analyzing additional plasma samples obtained at a single time point from 6 additional male COVID-19 patients between 5 and 12 days after onset of symptoms (Supplemental Table 7). Indeed, several of the cross-reactive anti-S, anti-N, and anti-pp1a antibodies that we had found in the female COVID-19 patient with severe ARDS were also detectable in male subjects and earlier in the course of infection (Supplemental Table 8).

## Discussion

Our comprehensive and systematic screen for antiviral antibody repertoires across individuals in 3 human cohorts revealed a large number of peptides with novel linear B cell epitopes in several proteins of endemic HCoVs. This is not surprising given that epidemic CoVs, and in particular SARS-CoV, have been the primary focus of previous immunological and epitope screening studies (34, 42). Information about the targets of immune responses to CoVs across different species provides a valuable resource for the prediction of candidate targets of newly emerging CoVs, as recently shown by Grifoni et al. (42). The authors were able to identify a priori several specific regions of the S, M, and N proteins of SARS-CoV-2 on the basis of sequence homology to the SARS-CoV virus, which are orthologous to several of the immunodominant regions of endemic HCoVs we identified. We detected antibodies against the structural S, N, M, and ORF8 proteins, as well as the nonstructural pp1ab polyprotein of HCoVs, the latter resembling the precursor for the large viral replicase complex (43). Interestingly, in another independent study, Grifoni et al. (28) recently reported similarly broad T cell responses in COVID-19 patients by employing an analogous screen for T cell epitopes of SARS-CoV-2 proteins using peptide ‘‘megapools’’ in combination with ex vivo T cell assays.

Surprisingly, circulating IgG antibodies in children appear to be differentially targeting structural and nonstructural proteins of HCoVs in comparison with adults (Figure 2). Whereas antibody specificities more frequently found in samples of pediatric subjects targeted structural proteins such as the S, N, and M proteins, in adult donors, a region of the nonstructural polyprotein pp1ab containing an acidic tandem repeat (NDDE[D/H]VVTGD) in HCoV-HKU1 appeared to be the predominant target of IgG antibodies. The latter polyprotein is posttranslationally processed into up to 16 subunits that form a large viral replicase complex; however, the function of the acidic tandem repeat and its role in pathogenesis remains unknown (43). This qualitative difference in the antibody repertoires of adult versus pediatric subjects appeared to be a specific characteristic of natural HCoV infection, as we did not find the same degree of variance in the antibody repertoires specific to other common respiratory viruses when comparing our cohorts and different age groups (Supplemental Figure 4). We speculate that the qualitative differences in antibody repertoires of adults versus children reflect a higher frequency and/or more recent exposure of children to seasonal coronaviruses than adults, coupled with the rapid decay of circulating anti-CoV antibodies that target the structural proteins of the virions. Further studies will be needed to fully understand the dynamics of antibody responses to endemic HCoVs.

Evidence for the transient and dynamic nature of humoral immunity to endemic HCoVs has been provided by numerous human serological studies, although many were conducted with only limited subjects, or only for selected species, and a variety of antibody detection methods were used that are not readily comparable (44–51). Nevertheless, evidence suggests that a sizable proportion of children experience primary infection with endemic HCoVs during their first year of life, and nearly all children have encountered at least one of the endemic HCoVs before 2 years of age, indicating that first exposure to endemic HCoVs occurs very early in life, similar to other common respiratory viruses, such as HRSV or HRVs (45, 49, 50). However, reported seroprevalence rates in older children and adults vary greatly depending on a variety of factors, including age and viral species. There is a general trend indicating that humoral immunity from primary infection with endemic HCoVs wanes quickly and that antibodies detected in older children and adults are rather a consequence of more recent reinfections (44–48). We estimated the seroprevalence of antibodies against endemic HCoVs to range between approximately 4% and approximately 27% depending on the species and cohort (Figure 1A). Given that endemic HCoV infections are common and usually acquired during early childhood (45, 46, 49, 50), it is likely that not only the adult subjects, but also many (if not all) of the children aged 7 to 15 years that were assessed in our study, have already experienced multiple infections with endemic HCoVs in their lifetime. Therefore, our estimated seroprevalence rates likely reflect the complex dynamics between rates of (re-)infection and waning humoral immunity over time. In agreement with this notion, age was negatively associated with seroprevalence in our study, suggesting that the duration of immunity in response to natural infection with endemic HCoVs and/or rates of reinfection decrease with increasing age. The dynamics of humoral immunity from past CoV infections is best described in studies of MERS and SARS patients. Although limited in sample size, these studies have shown that antibody titers in all previously infected individuals decline relatively quickly to minimally detectable levels over 2 to 3 years and that patients who suffered from more severe disease had higher and longer lasting total binding antibody titers and neutralizing titers (51). There is also evidence that symptomatic COVID-19 patients mount robust antibody responses that wane quickly over 6 months (52). However, the same study suggested that SARS-CoV-2–specific memory B cells may be sustained over a longer period (52). Indeed, most acute virus infections induce some level of protective and long-term immunity, albeit through a variety of mechanisms that are not necessarily the same for each pathogen and may even differ between hosts due to a variety of factors, including simultaneous viral coinfection (53, 54). Interestingly, a recent study by Weisberg et al. (55) demonstrated distinct antibody responses to SARS-CoV-2 in children and adults, which were found to be independent of the clinical outcomes and severity of infection. Even children with mild disease generated antibody responses against SARS-CoV-2 with reduced breadth and surprisingly also reduced neutralizing activity compared with adults. It is therefore also possible that children experience an altogether distinct course of infection compared with adults, and consequently differ in their serological responses, perhaps due to differences in expression levels of the viral receptor, angiotensin-converting enzyme 2, in airway epithelial cells (55). This requires further research. We also found a marginal but significant positive association between seroprevalence of endemic HCoVs and male sex (Figure 1B), which is consistent with an earlier report by Gaunt et al. (5).

Despite the variable degree of sequence conservation among different CoV species, the results of our systematic antibody screen highlight that the structural proteins of the virions share common antigenic sites. Indeed, several of the immunodominant regions we have identified experimentally in the structural proteins of endemic HCoVs are orthologous to the regions thought to be immunodominant targets for immune responses to SARS-CoV-2 (42) (Supplemental Figure 8), including 2 linear epitopes on the SARS-CoV-2 S protein that elicit potent neutralizing antibodies in COVID-19 patients (56). Importantly, antigenic regions that we found to be immunodominant in our study (i.e., enriched in ≥1% of all samples), as well as those corresponding to peptides for which enrichment was strongly and significantly associated with pediatric subjects (OR ≥ 2; *P* ≤ 0.005, Fisher’s exact test), mapped to functionally important regions of the structural CoV proteins. These included regions for receptor binding and the proteolytic cleavage sites of the S protein, as well as the N-terminal RNA-binding and C-terminal dimerization domains of the N protein, which have been shown to be critical for virus attachment and entry, cell-to-cell fusion, and virus replication (41, 57–61). The region of the S1 subunit responsible for receptor binding differs considerably among CoV species, which utilize different domains and host cell receptors and consequently differ in their tissue tropism (37–40, 46, 62). However, the S2 subunit resembling the fusion machinery is more conserved, both structurally and in amino acid sequence (38, 63) (Supplemental Figure 6). Indeed, we identified an immunodominant and highly conserved linear epitope immediately downstream of the furin-like S2′ cleavage site of the S protein (R¯SA[I/L]ED[I/L]LF) that likely resembles the fusion peptide, although its precise location has been disputed (64). The same antigenic site has recently been found on the SARS-CoV-2 S protein to elicit neutralizing antibodies in COVID-19 patients (56). The high degree of amino acid sequence and conformational conservation of the α-helical region immediately adjacent to the S2′ cleavage site (Figure 3E and Supplemental Figure 6A) likely explains why antibodies targeting this region also cross-reacted with orthologous peptides of related CoVs in our study, including those of MERS-CoV, SARS-CoV, and nonhuman isolates, further supporting our overall hypothesis and the important role of this particular region as a pan-CoV target site (40, 56). It is therefore tempting to speculate that at least in some individuals, past infections with endemic HCoVs have elicited cross-reactive antibodies and/or led to the generation of longer lived memory B cells with specific reactivity to this linear epitope, which may provide cross-protection against MERS or COVID-19. This may be the case particularly among children, who are generally less likely to experience severe disease outcomes from infection with epidemic CoVs (17–19, 21). In this context, it is important to highlight that antiviral antibodies can have a variety of protective effector functions that operate through different mechanisms. The underlying mechanisms for antibody-dependent neutralization of enveloped viruses (i.e., the inhibition of virus replication by blocking viral entry into the host cell) include the competitive binding of high-affinity antibodies — via their variable fragment antigen-binding regions — to specific regions within the viral attachment and fusion protein(s) that are also critical for the interaction with the host cell receptor(s) or activating host proteases (56). Neutralizing antibodies may also interfere with the fusion machinery, which undergoes profound activating conformational changes upon viral attachment to overcome the repulsive force between the viral envelope and host cell membrane bilayers (38). Additional antibody effector functions are Fc mediated, require the participation of additional host immune components, and are not necessarily restricted to antibodies targeting the viral attachment and fusion protein(s). This includes complement-dependent cytotoxicity, as well as enhancement of antibody-dependent cell-mediated cytotoxicity and/or phagocytosis (65, 66). The specific effector functions and mechanisms that are primarily responsible for the generally milder clinical outcome among children when infected with epidemic CoVs remain elusive (55).

Broadly cross-reactive antibody responses are also known for other enveloped RNA viruses, which may positively or negatively affect subsequent infection or vaccination. Flaviviruses, for example, are antigenically related, and broadly flavivirus cross-reactive antibodies from previous yellow fever vaccination have been shown to impair and modulate immune responses to tick-borne encephalitis vaccination (67). Similarly, immune history has been shown to profoundly affect protective B cell responses to influenza (68). Since we detected pan-CoV cross-reactive antibodies less frequently in plasma samples from adult donors, our results argue against a strong therapeutic benefit of intravenous immunoglobulin products to control the spread of COVID-19 disease (69). In this context, it should be noted that large-scale antibody screening by PhIP-Seq may frequently fail to detect conformational and posttranslationally modified B cell epitopes (29). Nonetheless, we found anti-CoV antibodies in plasma of a COVID-19 patient after prolonged hospitalization and intensive care that targeted largely the same structurally conserved and functionally important regions of the viral N and S proteins (Supplemental Figure 8) as those that we detected in a sizable proportion of children, including antibodies binding to the highly conserved motif and furin-like S2′ cleavage site (R¯SA[I/L]ED[I/L]LF), which provides further evidence for the clinical benefit of using convalescent plasma for the prevention and treatment of COVID-19 (65, 70, 71).

Our findings may also have important implications for the development of prophylactic or therapeutic monoclonal antibodies and vaccine design, such as in the context of COVID-19 (42, 72). The design of immunogens for next-generation vaccines and the development of monoclonal antibody therapies requires a detailed understanding of the immunogen structure and antibody recognition sites. Endemic HCoVs share common features with epidemic human-tropic CoVs and other enveloped, human-pathogenic viruses, many of which remain obscured by current amino acid sequence alignment tools due to the rapid evolution of viruses. The attachment and fusion protein(s) of enveloped viruses, for example, are key immunogens that share common structural features and employ a similar mechanism for catalyzing membrane fusion between the viral envelope and host cell. The coronavirus S protein belongs to the so-called class I viral fusion proteins, along with the influenza hemagglutinin protein, the HRSV fusion (F) protein, the Ebola virus glycoprotein, and the HIV-1 envelope (Env) protein. An important characteristic of these proteins is their conformational dynamics, which is critical for their function, but this has also proven a major challenge for structural analyses. Studies of HIV Env, HRSV F, and the F proteins of other enveloped viruses have highlighted that potent neutralizing antibodies primarily recognize the protein’s prefusion form in the closed conformation and that it is important to stabilize this form for structural analysis (in some cases in complex with bound antibodies), as well as for immunogen design to avoid undesirable antibody responses (38).

## Methods

### Study design and samples.

We performed a retrospective analysis of deidentified or coded plasma and serum samples collected from 3 human cohorts, namely: (a) 400 healthy male ABD of a blood bank in Qatar with diverse ethnic backgrounds and nationalities (Supplemental Figure 1A); (b) 800 adult male and female Qatari nationals and long-term residents of Qatar who are participating in a national cohort study — the QBB — and who represent the local population in the State of Qatar (31); and (c) 231 pediatric subjects with Qatari nationality who were admitted to, or visited outpatient clinics of, Sidra Medicine. Leftover plasma samples from healthy blood bank donors were collected from 2012 to 2016, deidentified, and stored at –80°C. For this study, specimens from male Qatari nationals 19 to 66 years of age (Supplemental Table 1) were selected from a larger blood donor cohort including 5983 individuals, and then age-matched male donors with other nationalities were randomly selected (Supplemental Figure 1). Samples from female blood bank donors were excluded because they were largely underrepresented in the blood bank donor cohort. We also excluded samples for which age, sex, or nationality information was lacking. Serum samples from the QBB cohort were collected from 2012 to 2017 and were randomly selected samples from the first 3000 individuals taking part in a longitudinal cohort study as described previously (31). Plasma samples from pediatric patients were selected from leftovers of samples processed in the clinical chemistry labs of Sidra Medicine, a tertiary care hospital for children and women in Doha, Qatar, from September to November 2019. In order to select appropriate pediatric samples, electronic medical records were queried using Discern analytics to identify blood samples from Qatari nationals aged 7 to 15 years for whom basic metabolic panel and comprehensive metabolic panel testing were done in the previous week. Samples from oncology patients, patients requiring complex care, and those in intensive care units, as well as samples from patients with chronic diseases, samples with no centile data, and samples from patients who were underweight (centile < 5%) or overweight were excluded. However, we included obese patients in our analysis, since a considerable proportion of Qatari nationals are overweight. One of the COVID-19 patients assessed in this study was a previously healthy female Belgian national with autosomal recessive interferon regulatory factor 7 deficiency who developed ARDS following SARS-CoV-2 infection at the age of 49 (73). For comparison, we also assessed unexposed family members, including the father, mother, brother (heterozygous carriers), and WT sister, as well as an unrelated age- and sex-matched healthy control. Additional male COVID-19 patients assessed in this study were between 30 and 68 years of age and residents of the State of Qatar with diverse nationalities (Supplemental Table 7). All patients assessed here required intensive care for COVID-19; however, information about preexisting comorbidities among the latter patients was not obtained.

### Phage immunoprecipitation-sequencing.

The VirScan phage library used for PhIP-Seq in the present study had been obtained from Stephen Elledge (Brigham and Women’s Hospital and Harvard Medical School, Boston, Massachusetts, USA). Large-scale serological profiling of the antiviral IgG repertoires in the individual serum or plasma samples was performed as described by Xu et al. (30). Each serum or plasma sample was tested in duplicate, and samples were analyzed in batches with up to 96 samples per batch. Only samples that satisfied a minimum read count of 1 × 10^6^ as well as a Pearson correlation coefficient of at least 0.7 in the 2 technical repeats were considered for downstream analysis. Data from 30 individuals of the ABD cohort and 2 individuals of the QBB cohort were excluded from the downstream analysis because of insufficient sequencing read depth, of low sequencing data quality, or 1 of 2 technical replicates had failed (data not shown).

### Peptide enrichment analysis.

To filter for enriched peptides, we first computed −log_10_*P* values as described previously (30, 32, 35) by fitting a 0-inflated generalized Poisson model to the distribution of output counts and regressed the parameters for each peptide sequence based on the input read count. We considered a peptide enriched if it passed a reproducibility threshold of 2.3 (−log_10_*P*) in 2 technical sample replicates. To remove sporadic hits, we then filtered for antibody specificities to CoV peptides that were found to be enriched in at least 3 of all 1399 subjects assayed and analyzed in this study. We computed species-specific significance cutoff values to estimate the minimum number of enriched, nonhomologous peptides required to consider a sample seropositive using a generalized linear model and in-house serological (ELISA) data from pooled samples that were tested positive for various viruses. We then computed virus score values as described by Xu et al. (30) by counting enriched, nonhomologous peptides for a given species and then adjusted these score values by dividing them with the estimated score cutoff. For this study, we considered a peptide immunodominant if it was enriched in at least 1% of the samples obtained from the 3 larger cohorts (*n* = 1399) assayed and analyzed in this study.

### Association studies and differential enrichment analysis.

We applied a generalized linear model to test for associations between the HCoV species-specific adjusted score values, sex, and age. We considered an association significant if the *P* value was less than or equal to 0.001. We examined the frequency distribution of enriched peptides among samples of the different age groups (PED versus ABD + QBB) by estimating OR (reported as lod) and associated *P* values using Fisher’s exact test. Peptides that satisfied both significance (*P* ≤ 0.005) and magnitude criteria (|OR| ≥ 2) were considered differentially enriched. Positive OR and lod values indicated more frequent peptide enrichment among pediatric study subjects, whereas negative OR and lod values indicated more frequent peptide enrichment among adult subjects.

### Clustering of peptides for shared linear B cell epitopes.

To estimate the minimum number of linear B cell epitopes among the enriched peptides, we built a pairwise distance matrix that captured the maximum size of linear sequence identity of amino acids (d_i,j_) between all enriched peptides. Groups of peptides that shared ≥ 7 amino acids linear sequence identity (d_i,j_ ≥ 7) were assigned to a cluster. Peptides of a given cluster were considered to share a linear B cell epitope (Supplemental Figure 9).

### Software.

For statistical analyses and principal component analysis, we used open-source Python modules detailed below. Multiple sequence alignments were done using the MAFFT (74, 75) via EMBL-EBI’s web services and Java Alignment Viewer (Jalview) for visualization (76). Network analysis of peptide clusters (Figure 5 and Supplemental Figure 9) was performed using Python module NetworkX (version 2.5). Linear B cell epitopes were predicted using BepiPred-2.0 (77). Protein structures graphics were generated using PyMOL (Schrödinger).

### Data and code availability.

All data are available in the manuscript or the supplemental materials. Raw PhIP-Seq reads and Python in-house scripts used in this study are readily available upon request. The pipeline for processing the PhIP-Seq data has been published previously (29).

### Statistics.

All statistical analyses were performed with Python (version 3.6) using open-source modules, such as SciPy (version 1.14.1), Scikit-learn (version 0.23), Statsmodels (version 0.11), and in-house scripts. Antibody prevalence values for different species (Figure 1A, Supplemental Figure 4A, and Supplemental Table 5) were determined by dividing the number of seropositive individuals of a given group (i.e., cohort, age group, or sex) by the total number of samples of the respective group; calculated values were reported as percentage. For differential enrichment analysis of peptides in pediatric versus adult subjects (Figure 2B, Table 1, and Supplemental Table 6), we used Fisher’s exact test and accounted for multiple testing by Bonferroni’s correction. *P* values less than 0.005 were considered statistically significant. For association studies (Figure 1B), we used Student’s *t* test (2 tailed) and Bonferroni’s correction and considered associations with *P* values less than 0.001 statistically significant.

### Study approval.

The human subject research described here had been approved by the institutional research ethics boards of Sidra Medicine, Qatar Biobank, INSERM, Erasme Hospital, Hamad Medical Corporation, and Qatar University, depending on where subjects were recruited and research was carried out. This included the receipt of written informed consent from participants.

## Author contributions

TK and NM conceived the original idea, designed the models and the computational framework of the study, analyzed the data, and wrote manuscript. MR, FA, SSYH, MA, QZ, PB, ZL, EJ, VB, AC, HMY, MKS, LA, and JLC planned or performed the experiments. GKN, AS, IV, JCG, GS, I Migeotte, FH, I Meyts, and MRH contributed samples and data. MR, JLC, and MRH provided critical feedback to this version of the manuscript. All authors have seen and approved the manuscript. It has not been accepted or published elsewhere.

## Supplementary Material

Supplemental data

Supplemental Tables 3-4

## Figures and Tables

**Table 1:**
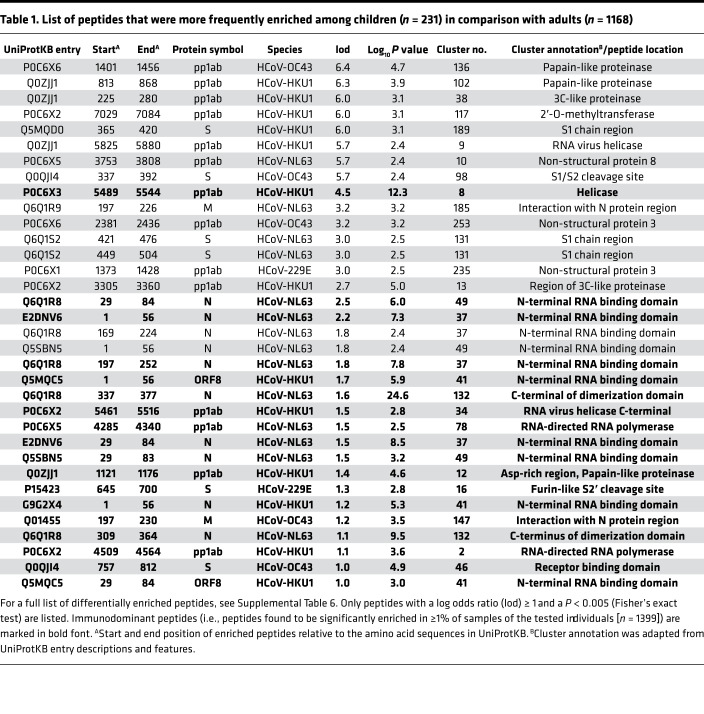
List of peptides that were more frequently enriched among children (*n* = 231) in comparison with adults (*n* = 1168)

## Version 1 01/26/2021

In-Press Preview

## Version 2 02/22/2021

Electronic publication
