# Supplementary material for: Distinct antibody repertoires against endemic human coronaviruses in children and adults
Source: JCI Insight. 2021 Feb 22;6(4):e144499. doi: 10.1172/jci.insight.144499 (PMC7934927; doi:10.1172/jci.insight.144499)
Supplement: Supplemental data [file jciinsight-6-144499-s069.pdf]

Supplemental Materials for

Distinct antibody repertoires against endemic human coronaviruses in children and adults

Taushif Khan, Mahbuba Rahman, Fatima Al Ali, Susie S. Y. Huang, Manar Ata, Qian Zhang, Paul Bastard, Zhiyong Liu, Emmanuelle Jouanguy Vivien Béziat, Aurélie Cobat, Gheyath K. Nasrallah, Hadi M. Yassine, Maria K. Smatti, Amira Saeed, Isabelle Vandernoot, Jean-Christophe Goffard, Guillaume Smits, Isabelle Migeotte, Filomeen Haerynck, Isabelle Meyts, Laurent Abel, Jean-Laurent Casanova, Mohammad R. Hasan, Nico Marr*

JCI Insight (February 3, 2021) version

*Corresponding author: Nico Marr, Sidra Medicine, Research Branch, Al Gharrafa Street, Ar-Rayyan, PO BOX 26999, Doha, Qatar, E-mail: nmarr@sidra.org

Content#
Supplemental Figures 1-9
Supplemental Tables 1, 2, 5-8

#Supplemental Tables 3 and 4 are provided as spreadsheets in a separate file.

Supplemental Figures

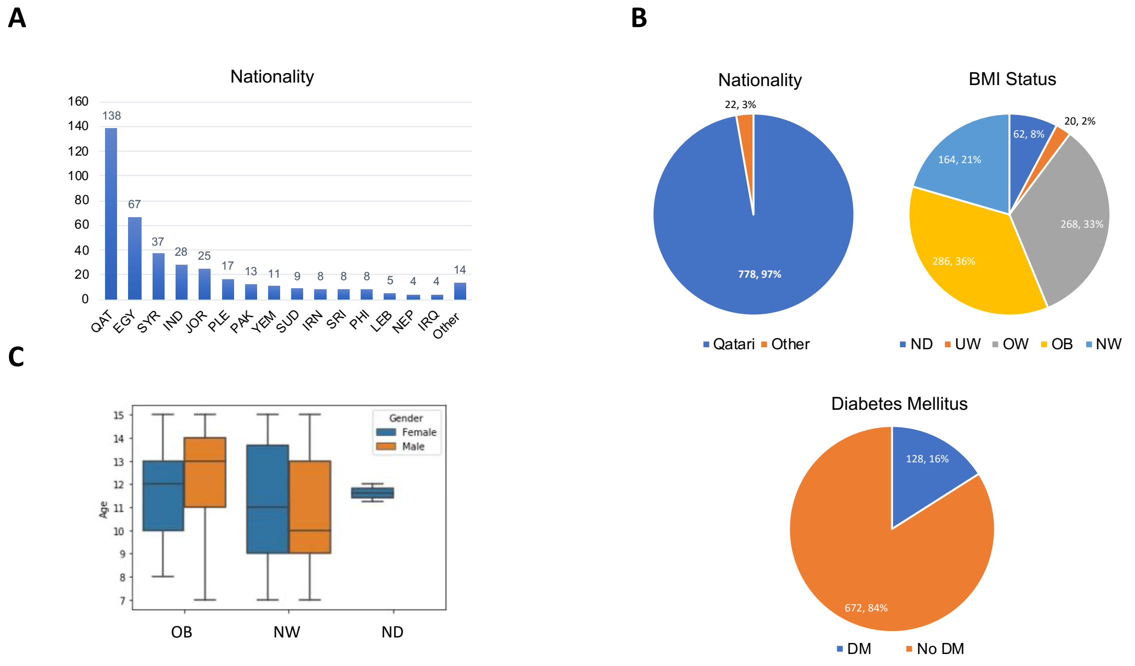

Supplemental Figure 1. Demographic and clinical features of the different human cohorts. A, Nationalities among the healthy blood bank donors. **B,** Number and fraction of Qatari nationals and long-term residents with other nationality comprising the 800 assayed individuals of the Qatar Biobank cohort, stratified by body mass index (BMI) and proportion of subjects with diabetes mellitus (DM). UW, underweight (BMI < 18); NW, normal weight (BMI ≥ 18 and < 25); OW, overweight (BMI ≥ 25 and <30); OB, obese (BMI ≥ 30). **C,** Number and fraction of male and female pediatric study subjects with normal weight (NW) or who were obese (OB). ND, not determined; QAR, Qatar; EGY, Egypt; SYR, Syria; IND, India; JOR, Jordan; PAK, Pakistan; YEM, Yemen; SUD, Sudan; IRN, Iran; SRI, Sri Lanka; PHI, Philippines; LEB, Lebanon; NEP, Nepal; IRQ, Iraq; Other, residents with other nationality; PLE, non-residents.

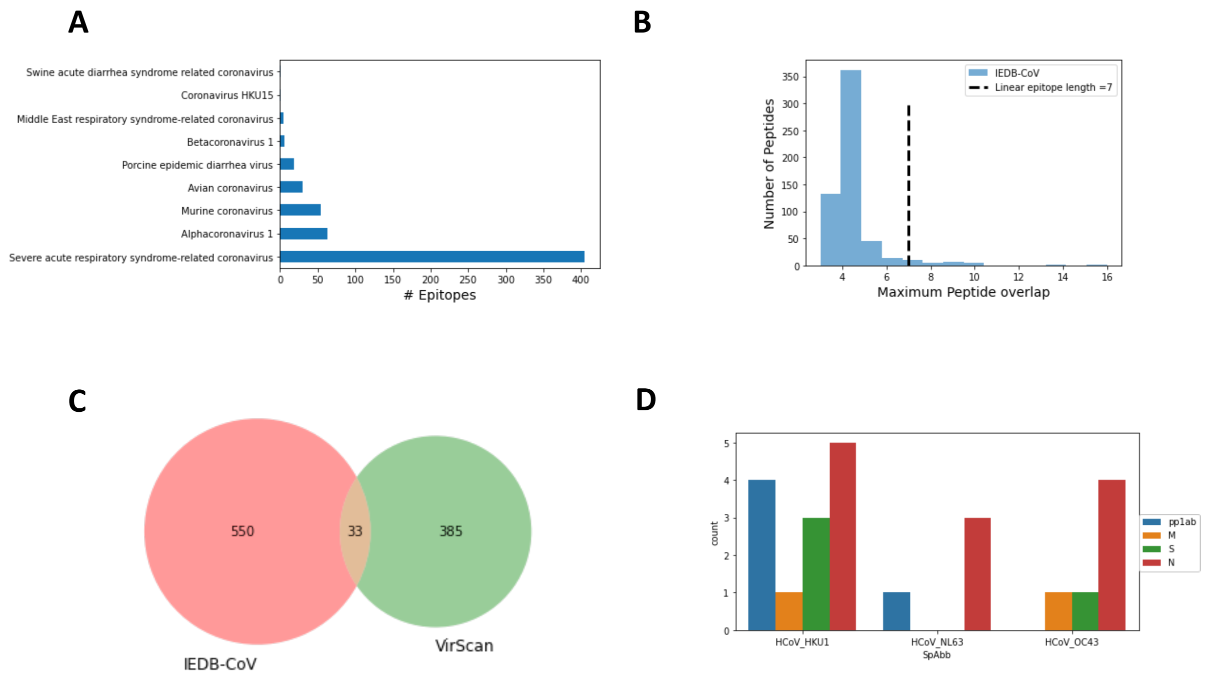

Supplemental Figure 2. Comparison analysis of enriched HCoV peptides identified by PhIP-Seq with B cell epitopes in the Immune Epitope Database (IEDB). A, Number of B cell epitopes for different CoV species available in IEDB (accessed and downloaded from www.iedb.org on 17 May 2020). **B**, Number of peptides from endemic HCoVs which we found to be significantly enriched in at least three of all samples (*N* = 1399) with homology in the linear amino acid sequence to 583 known CoV B cell epitopes in IEDB. We considered an epitope as a match if there was ≥ 7 linear sequence identity between query (IEDB epitope) and the amino acid sequence of the enriched HCoV peptide. **C,** Venn diagram depicting overlap between enriched peptides (i.e. matches) detected by VirScan and B cell epitopes from CoVs in IEDB. **D**, Number of matching peptides that share sequence homology with known B cell epitopes, stratified by species and viral protein.

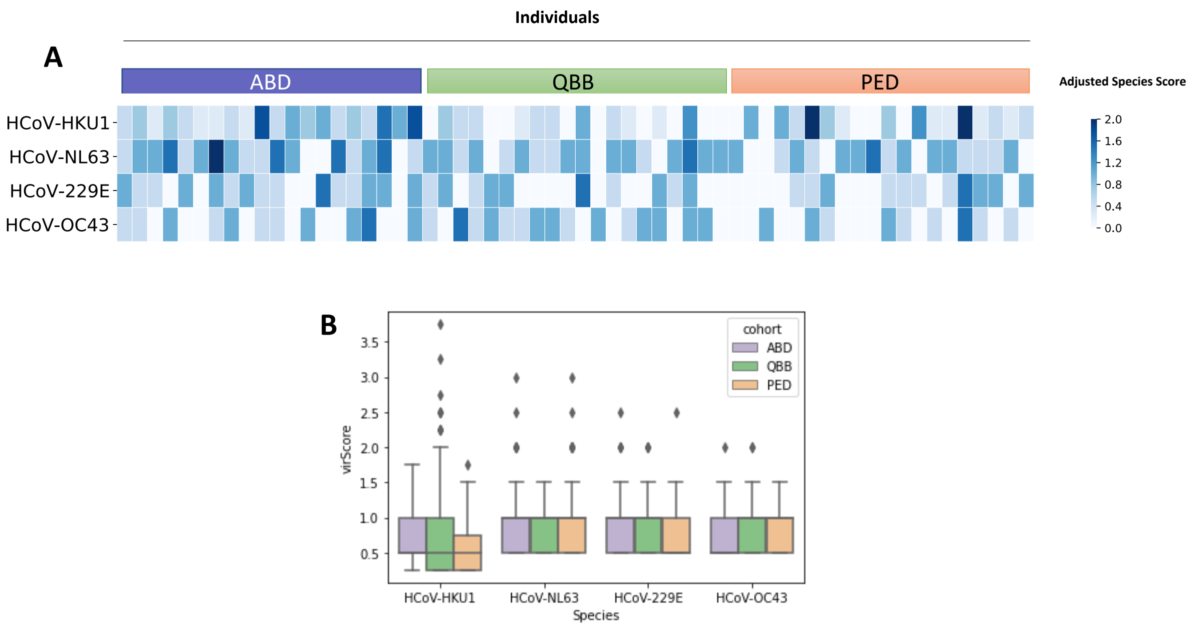

Supplemental Figure 3: Similar antibody repertoire breadth among seropositive individuals of the different cohorts. A, Heatmap plot depicting adjusted species score values for HCoV-HKU1, -NL63, -229E and -OC43 in randomly selected seropositive individuals (n = 20) of each cohort. Each individual is represented as a column. Values $\geq$ 1 indicate that the individual was seropositive for a given species. **B**, Species-wise variation in adjusted species score values among seropositive individuals of each cohort.

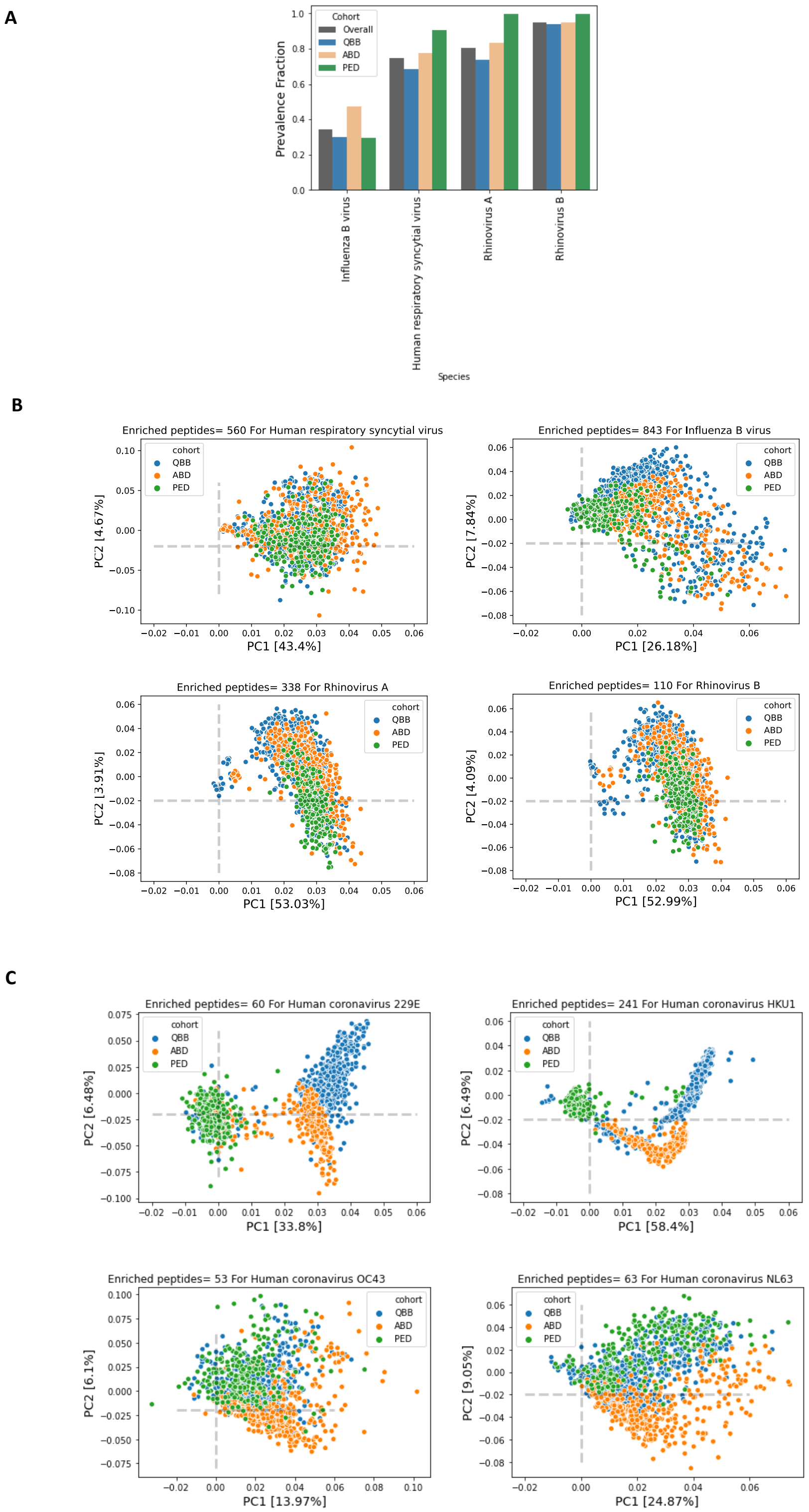

Supplemental Figure 4. Seroprevalence of respiratory viruses and variance in the antibody repertoires among individuals of the analyzed cohorts. A, Bar plot depicting the fraction of individuals found seropositive for human respiratory syncytial virus, rhinovirus A, rhinovirus B and influenza B virus among the tested subjects or by cohort. **B** and **C,** Principal component analysis of the antigenic peptides of human respiratory syncytial virus, rhinovirus A, rhinovirus B and influenza B virus **(B)**, as well as of human coronaviruses 229E, NL63, OC43 and HKU1 **(C)**, that we found to be enriched in at least 3 samples.

Supplemental Figure 5A

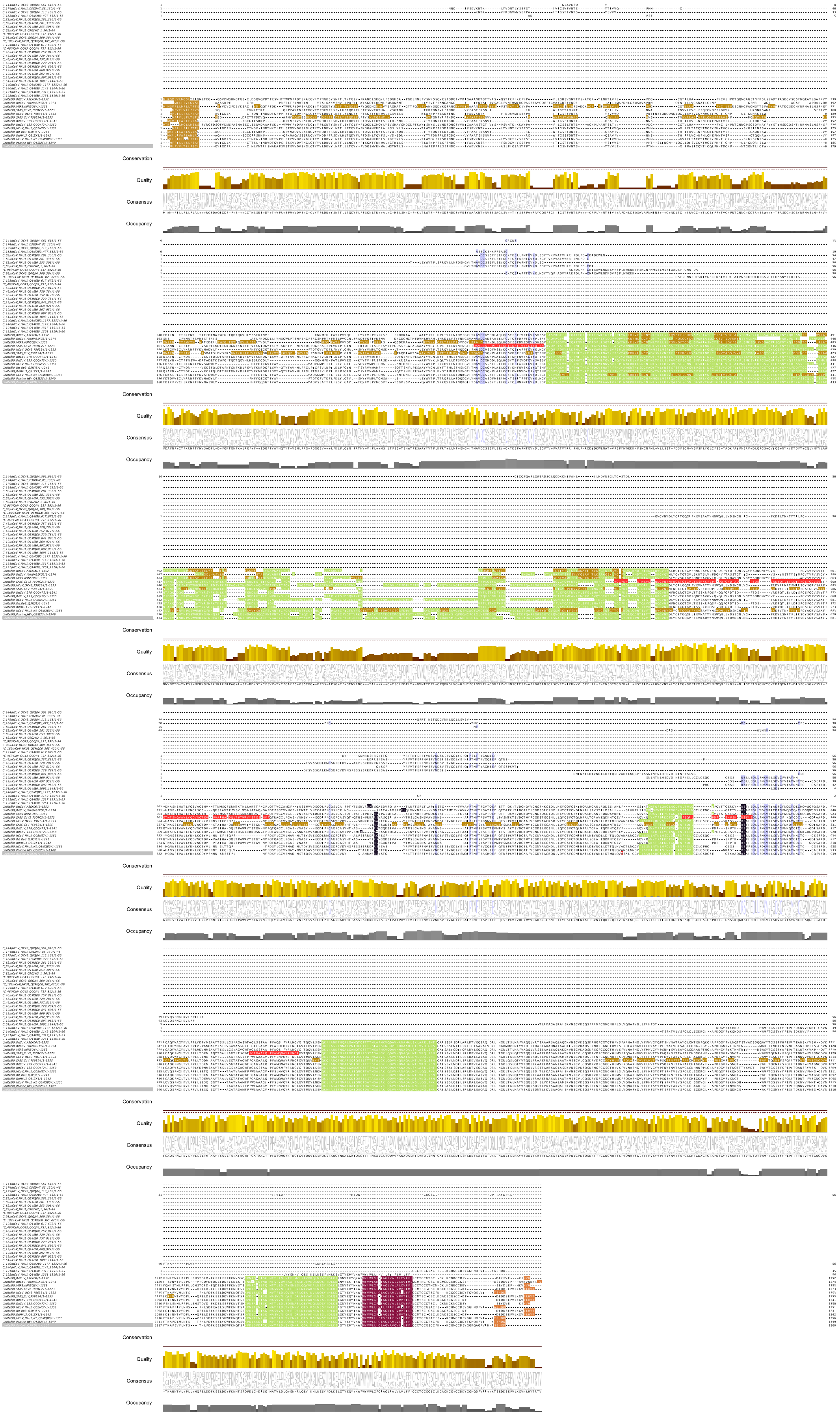

Supplemental Figure 5B

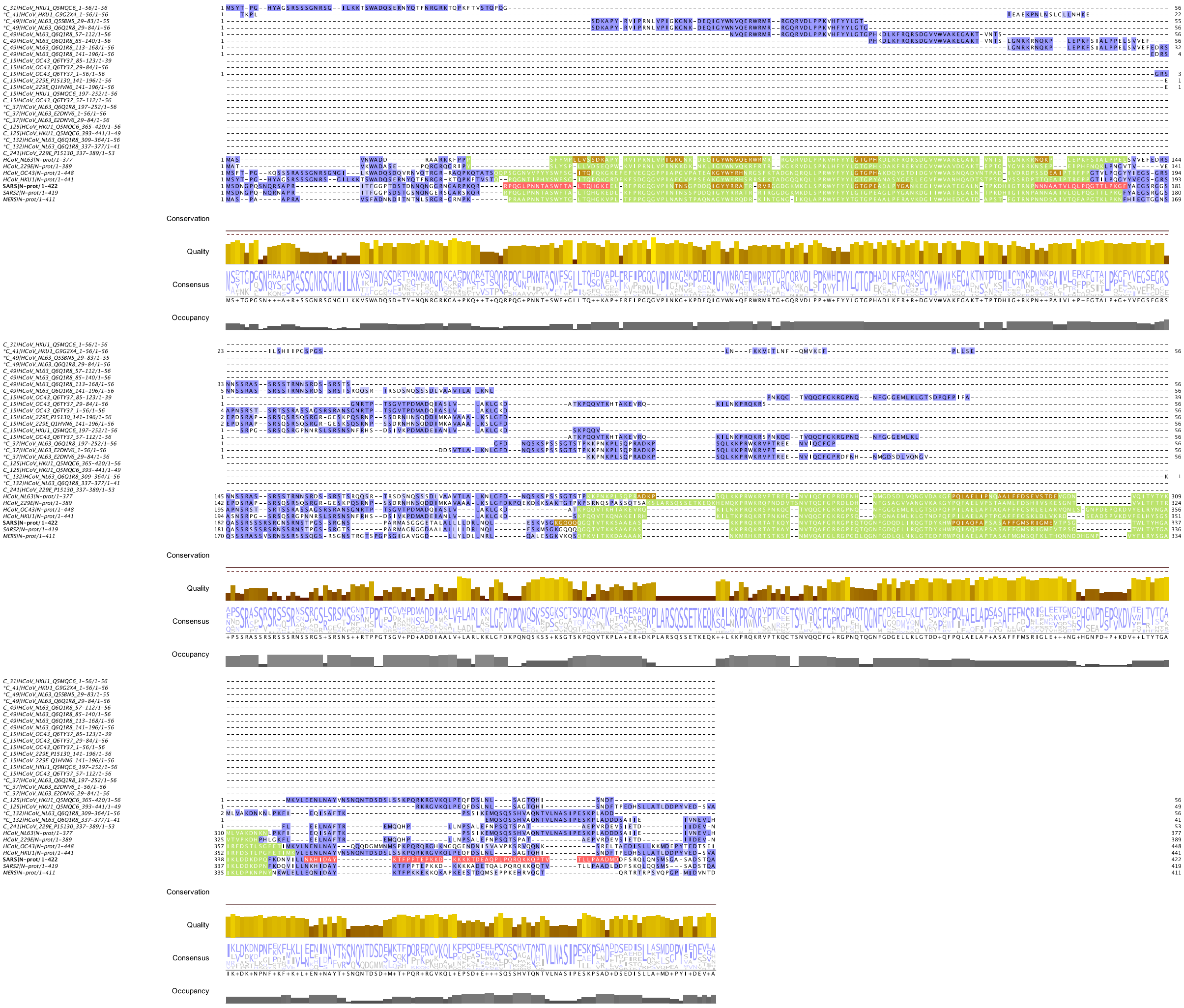

Supplemental Figure 5C

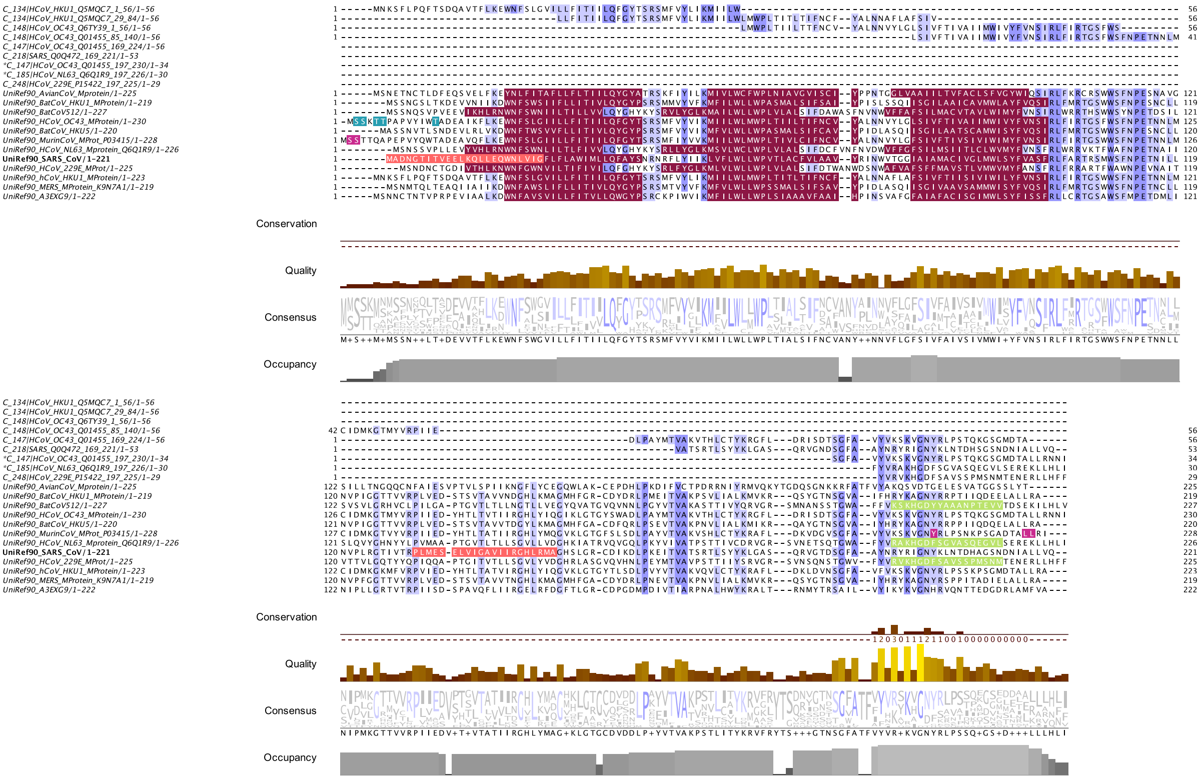

Supplemental Figure 5D

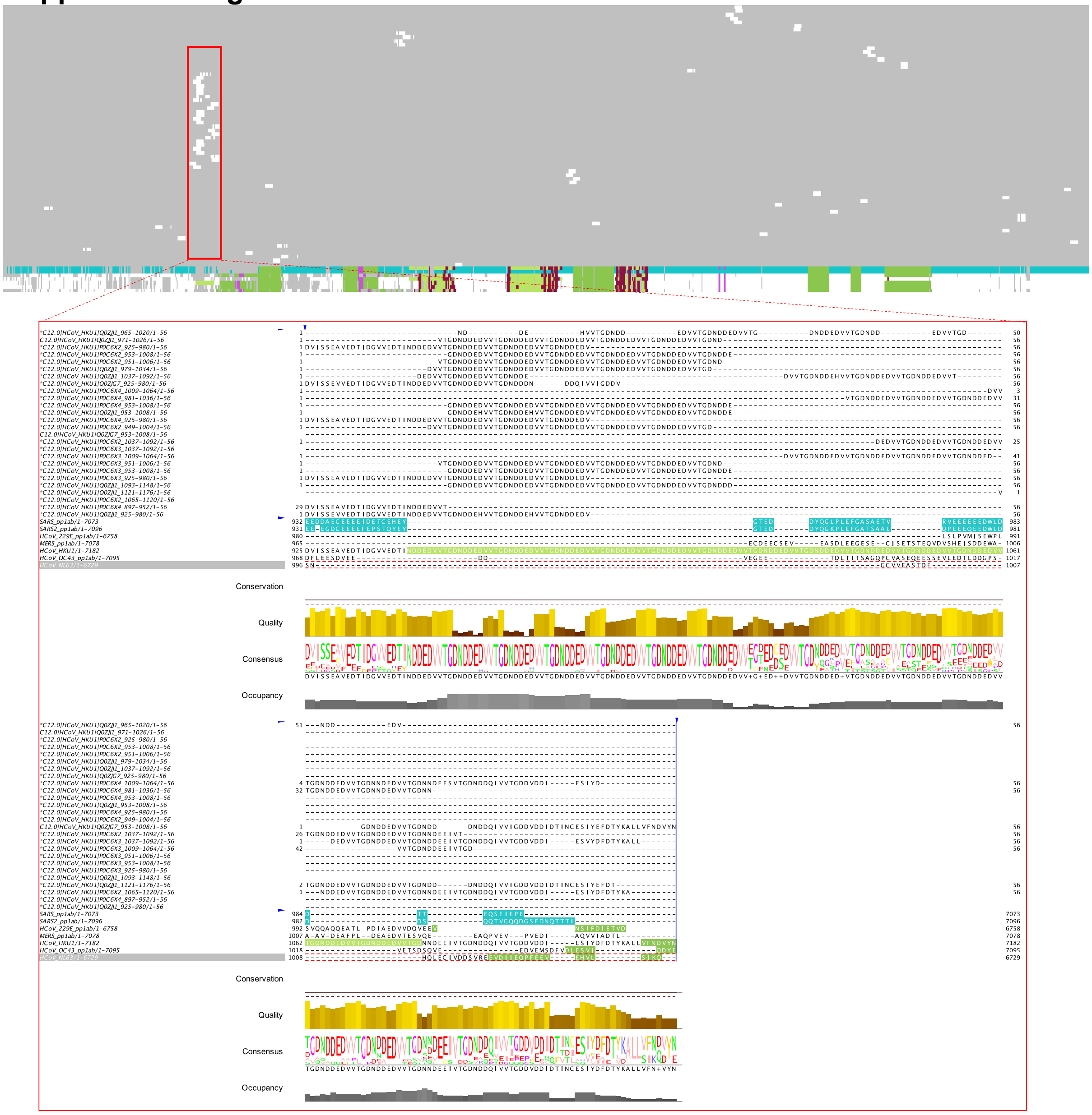

Supplemental Figure 5. Mapping of enriched, immunodominant peptides of endemic HCoVs to the full-length protein sequences. **A-D**, Multiple sequence alignments of the enriched, immunodominant peptides with the full-length sequences of the spike protein **(A)**, nucleocapsid protein **(B)**, matrix glycoprotein **(C)** and the Orf1ab replicase polyprotein **(D)** of selected coronaviruses. Amino acids with a high percentage of sequence identity are shown in violet. Reference sequence features are color-coded in accordance with the annotation and features available in UniProtKB and visualized using JalView. Green indicates regions of interest, red indicates transmembrane regions, orange indicates beta strands and pink indicates residues annotated as epitopes in IEDB. The cleavage sites (S1/S2 and S2') of the SARS-CoV S protein are shaded in black. For pp1ab, the sequence alignment is shown as an overview with gaps are shaded in grey and sequences are in white. Only part of the sequence alignment is shown, which depicts the PL1-PRO cleavage product containing a tandem repeat of N-[DN]-D-E-D-V-V-T-G-DA in HCoV-HKU1 (isolate N1) (UniProtKB entry and position P0C6X2[945-1084]).

A

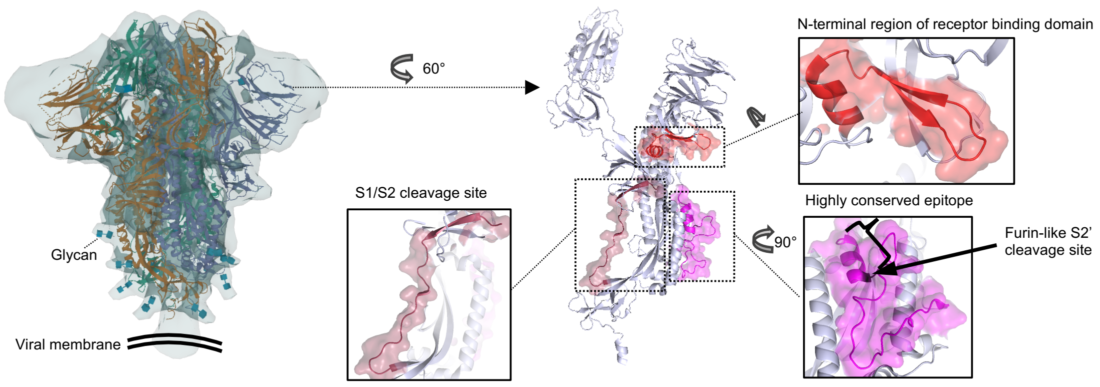

B

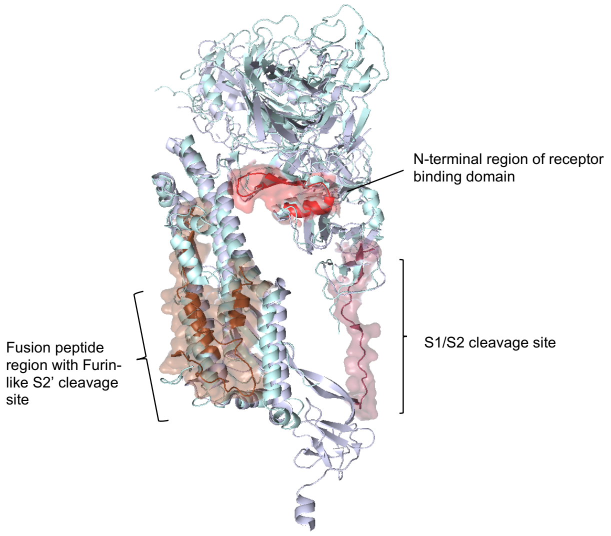

Supplemental Figure 6. Antigenic regions of the spike protein. A, Ribbon diagram of the SARS-CoV-2 S trimer with carbohydrate residues shown (left) and monomer without carbohydrate residues (middle) in the prefusion conformation (PDB id: 6VXX, chain A); regions for which multiple sequence alignments are shown in Figures 3C-E are depicted enlarged. **B,** Superimposition of the spike proteins of SARS-CoV-2 (6VXX) and HCoV-HKU1 (5I08) in the prefusion state. Root-mean-square deviation [rmsd] = 3.328 Ångström.

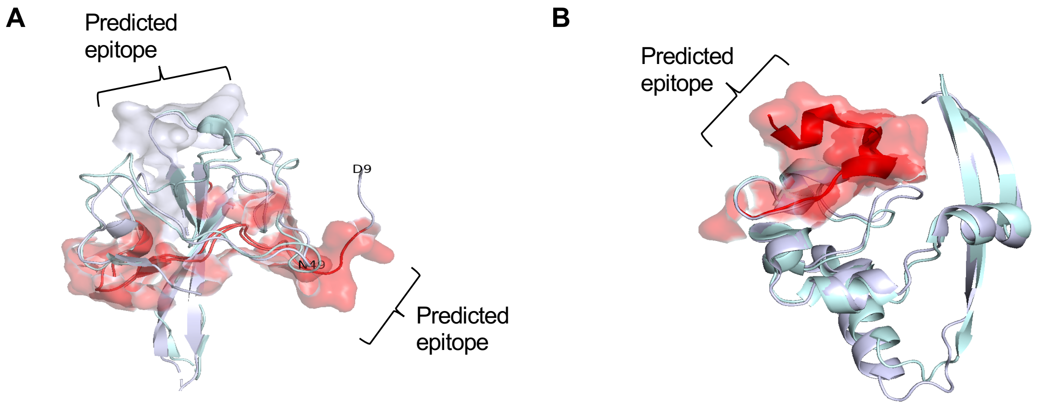

Supplemental Figure 7. Structurally conserved immunodominant regions of the nucleocapsid protein. A, Super-imposed ribbon structure of the N-terminal RNA-binding domain from HCoV-NL63 (PDB: 5N4k, chain A) and that from SARS-CoV-2 (PDB: 6M3M, chain A) (root-mean-square deviation [rmsd] = 0.7 Ångström). **B,** Super-imposed ribbon structure of the C-terminal self assembly domain of proteins from HCoV- NL63 (pdbId: 5EPW, chain A) and that of SARS-CoV-2 (pdbID: 6WJI, chain A) (rmsd = 0.91 Ångström).

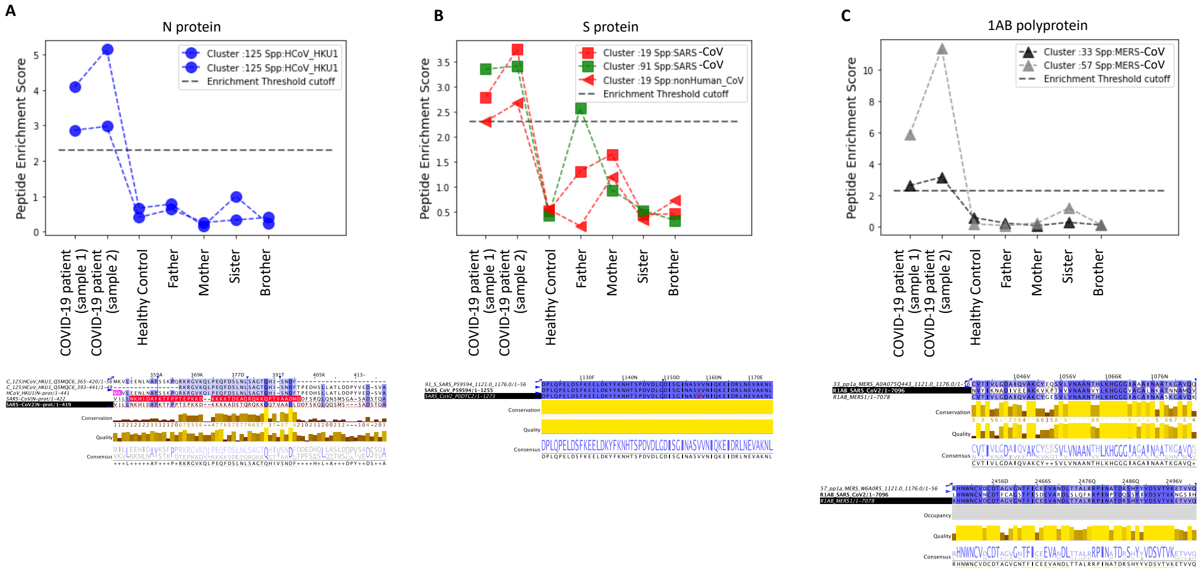

Supplemental Figure 8. Cross-reactive antibody responses in a COVID-19 patient with proteins of other coronaviruses. A-C, Plasma samples of an adult COVID-19 patient, unexposed family members (father, mother, sister and brother) as well as an unrelated healthy, age- and gender-matched control were subjected to PhIP-Seq analysis. Samples from the COVID-19 patient were obtained at two time points: during acute infection (sample 1) and during the convalescent phase, one week after hospital discharge (sample 2). Shown is a comparison of enrichment scores of peptides derived from the N protein **(A)**, S protein **(B)** and 1AB polyprotein **(C)** of various coronaviruses. HCoV-HKU1 peptides are depicted as circles, SARS-CoV peptides are depicted as squared symbols and MERS-CoV as well as non-human CoV peptides are shown as triangles. SARS-CoV-2 peptides were not included in the VirScan phage library used in our study. The dashed line indicates the significance cut-off for the peptide enrichment [$-\log10(P) \geq 2.3$]. The cluster numbers indicate linear epitopes as listed in Table S3. The multiple sequence alignments (bottom) of the enriched peptides with the reference sequences of SARS-CoV-2 and the respective CoV species show the degree of sequence identity across species. Known epitopes in the Immune Epitope Database (IEDB) are shown in pink. Sequences were colored to indicate percent sequence identity and visualized using Jalview.

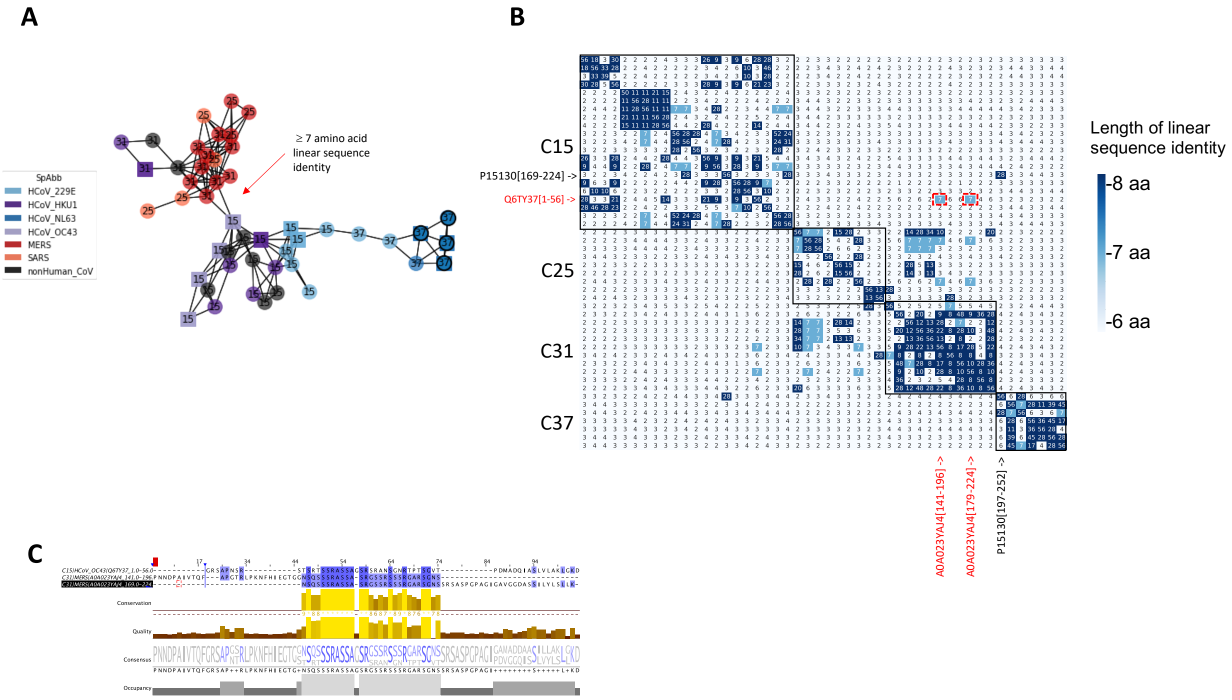

Supplemental Figure 9. Clustering of peptides for shared linear B cell epitopes. A, Network representation of peptides built from a pairwise distance matrix as shown in Figure 5B. Each node represents an enriched peptide and the color indicates the species. Edges indicate ≥ 7 amino acids linear sequence identity between two nodes (i.e. peptides), the estimated size of a linear B cell epitope. **B**, Pairwise distance matrix of peptides targeted by cross-reactive antibodies, including enriched peptides of clusters 15, 25, 31 and 37. The cluster assignment has been indicated as a black box. Distance pairs with ≥ 7 amino acid linear sequence identity are shown in dark blue. Distance pairs marked with a red box represent a 7 amino acid linear sequence identity between enriched peptides of more distantly related CoVs, namely HCoV-OC43 (Q6TY37[1-56]) and MERS-CoV (A0A023YAJ4[141-196] and A0A023YAJ4[179-224]). **C**, Multiple sequence alignment of enriched HCoV-OC43- and MERS-CoV-derived peptides Q6TY37[1-56], A0A023YAJ4[141-196] and A0A023YAJ4[179-224] encoding part of the N-terminal intrinsically disordered region (IDR) of the N protein. The alignment shows the 7 amino acid linear sequence identity in a low-complexity sequence motive (SSRASSA) which likely represents a broadly cross-reactive antibody binding site.

Supplemental Tables

Supplemental Table 1. Proteins of CoVs represented in the VirScan phage library

Species[A]	*Organism*	*Protein*	*No. of Peptides*
Alphacoronavirus 1	Feline coronavirus (strain FIPV WSU-79/1146) (FCoV)	NS	239
Bat coronavirus 1B	Bat coronavirus 1B	Rep1a	249
Betacoronavirus 1	Bovine coronavirus	N	4
	Bovine coronavirus (strain 98TXSF-110-ENT) (BCoV-ENT) (BCV)	E	2
		N	7
		NS	12
		S	48
	Equine coronavirus	Rep1a	254
	**Human coronavirus OC43 (HCoV-OC43)**	**HE**	**15**
		**M**	**11**
		**N**	**4**
		**NS**	**253**
		**S**	**37**
	Porcine hemagglutinating encephalomyelitis virus (strain 67N) (HEV-67N)	N	16
Human coronavirus 229E	**Human coronavirus 229E (HCoV-229E)**	**E**	**2**
		**M**	**8**
		**N**	**23**
		**NS**	**248**
		**ORF4**	**10**
		**Rep1b**	**2**
		**S**	**63**
Human coronavirus HKU1	**Human coronavirus HKU1 (HCoV-HKU1)**	**N**	**13**
		**Rep1ab**	**510**
		**S**	**15**
	**Human coronavirus HKU1 (isolate N1) (HCoV-HKU1)**	**E**	**2**
		**HE**	**13**
		**M**	**7**
		**N**	**22**
		**NS**	**259**
		**S**	**48**
	**Human coronavirus HKU1 (isolate N2) (HCoV-HKU1)**	**E**	**2**
		**HE**	**13**
		**N**	**7**
		**NS**	**258**
		**S**	**48**
	**Human coronavirus HKU1 (isolate N5) (HCoV-HKU1)**	**NS**	**254**
Human coronavirus NL63	**Human coronavirus NL63 (HCoV-NL63)**	**E**	**2**
		**M**	**10**
		**N**	**18**
		**NS**	**248**
		**ORF3**	**1**
		**Rep1a**	**1**
		**S**	**71**
Middle East respiratory syndrome coronavirus	BtVs-BetaCoV/SC2013	E	2
		M	7
		N	15
		ORF3	3
		ORF4	11
		ORF5	8
		ORF8	6
		Rep1a	256
		S	47
	Coronavirus Neoromicia/PML-PHE1/RSA/2011	E	2
		ORF3	3
		ORF4	12
		ORF5	7
		ORF8	7
		Rep1a	156
		S	47
	Human coronavirus EMC (isolate United Kingdom/H123990006/2012) (HCoV-EMC)	E	2
		M	7
		N	14
		NS	408
		ORF3	3
		ORF4	11
		ORF5	7
		S	48
	Middle East respiratory syndrome coronavirus	N	8
		ORF4	11
		ORF5	11
		ORF8	3
		Rep1a	1018
		Rep1ab	95
		Rep1b	63
		S	108
Severe acute respiratory syndrome-related coronavirus	Bat coronavirus 279/2005 (BtCoV) (BtCoV/279/2005)	E	2
		M	7
		NS	254
		U	2
	Human SARS coronavirus (SARS-CoV)	N	15
		NS	9
		ORF3	9
		ORF7	4
		ORF9	3
		S	44
	Human SARS coronavirus (isolate Tor2) (SARS-CoV)	Rep1b	1

[A]Proteins of endemic hCoVs are shown in bold.

Supplemental Table 2. Number of individuals included in the downstream analysis

Cohort	Gender	No. of subjects	Age (years)			
			Mean	Std	Min	Max
ABD	Male	370	40.1	10.1	19	66
PED	Female	117	11.4	2.2	7	15
	Male	114	11.8	2.3	7	15
QBB	Female	511	40.4	12.9	19	81
	Male	287	41.0	12.9	19	81

QBB, Qatar Biobank cohort; ABD, adult blood bank donors; PED, pediatric subjects.

Supplemental Table 5. Seroprevalence of endemic HCoVs stratified by gender and age group

Species	Age group	Gender	Prevalence (%)	Mean prevalence per age group (%)
HCoV-229E	adults	female	4.3	11.7
		male	19.0	
	children	female	10.3	8.2
		male	6.1	
HCoV-HKU1	adults	female	4.1	6.5
		male	8.8	
	children	female	7.7	6.5
		male	5.3	
HCoV-NL63	adults	female	9.2	13.0
		male	16.7	
	children	female	21.4	23.8
		male	26.3	
HCoV-OC43	adults	female	4.1	5.8
		male	7.5	
	children	female	14.5	12.5
		male	10.5	

The adult group (*n* = 1168) includes adult blood bank donors and individuals of the Qatar Biobank cohort.

Supplemental Table 6. List of peptides that were differentially enriched in children (*n* = 231) versus adults (*n* = 1168)

UniProtKB entry	Start[A]	End[A]	Protein symbol	Species	lod	Log_{10} *P* value	Cluster no.	Cluster annotation[B] / peptide location
P0C6X6	1401	1456	pp1ab	HCoV-OC43	6.4	4.7	136	Papain-like proteinase
Q0ZJJ1	813	868	pp1ab	HCoV-HKU1	6.3	3.9	102	Papain-like proteinase
Q0ZJJ1	225	280	pp1ab	HCoV-HKU1	6.0	3.1	38	3C-like proteinase
P0C6X2	7029	7084	pp1ab	HCoV-HKU1	6.0	3.1	117	2'-O-methyltransferase
Q5MQD0	365	420	S	HCoV-HKU1	6.0	3.1	189	S1 chain region
Q0ZJJ1	5825	5880	pp1ab	HCoV-HKU1	5.7	2.4	9	RNA virus helicase
P0C6X5	3753	3808	pp1ab	HCoV-NL63	5.7	2.4	10	Non-structural protein 8
Q0QJI4	337	392	S	HCoV-OC43	5.7	2.4	98	S1/S2 cleavage site
**P0C6X3**	**5489**	**5544**	**pp1ab**	**HCoV-HKU1**	**4.5**	**12.3**	**8**	**Helicase**
Q6Q1R9	197	226	M	HCoV-NL63	3.2	3.2	185	Interaction with N protein region
P0C6X6	2381	2436	pp1ab	HCoV-OC43	3.2	3.2	253	Non-structural protein 3
Q6Q1S2	421	476	S	HCoV-NL63	3.0	2.5	131	S1 chain region
Q6Q1S2	449	504	S	HCoV-NL63	3.0	2.5	131	S1 chain region
P0C6X1	1373	1428	pp1ab	HCoV-229E	3.0	2.5	235	Non-Structural protein 3
P0C6X2	3305	3360	pp1ab	HCoV-HKU1	2.7	5.0	13	Region of 3C-like proteinase
**Q6Q1R8**	**29**	**84**	**N**	**HCoV-NL63**	**2.5**	**6.0**	**49**	**N-terminal RNA binding domain**
**E2DNV6**	**1**	**56**	**N**	**HCoV-NL63**	**2.2**	**7.3**	**37**	**N-terminal RNA binding domain**
Q6Q1R8	169	224	N	HCoV-NL63	1.8	2.4	37	N-terminal RNA binding domain
Q5SBN5	1	56	N	HCoV-NL63	1.8	2.4	49	N-terminal RNA binding domain
**Q6Q1R8**	**197**	**252**	**N**	**HCoV-NL63**	**1.8**	**7.8**	**37**	**N-terminal RNA binding domain**
**Q5MQC5**	**1**	**56**	**ORF8**	**HCoV-HKU1**	**1.7**	**5.9**	**41**	**N-terminal RNA binding domain**
**Q6Q1R8**	**337**	**377**	**N**	**HCoV-NL63**	**1.6**	**24.6**	**132**	**C-terminal of dimerization domain**
**P0C6X2**	**5461**	**5516**	**pp1ab**	**HCoV-HKU1**	**1.5**	**2.8**	**34**	**RNA virus helicase C-terminal**
**P0C6X5**	**4285**	**4340**	**pp1ab**	**HCoV-NL63**	**1.5**	**2.5**	**78**	**RNA-directed RNA polymerase**
**E2DNV6**	**29**	**84**	**N**	**HCoV-NL63**	**1.5**	**8.5**	**37**	**N-terminal RNA binding domain**
**Q5SBN5**	**29**	**83**	**N**	**HCoV-NL63**	**1.5**	**3.2**	**49**	**N-terminal RNA binding domain**
**Q0ZJJ1**	**1121**	**1176**	**pp1ab**	**HCoV-HKU1**	**1.4**	**4.6**	**12**	**Asp-rich region, Papain-like proteinase**
**P15423**	**645**	**700**	**S**	**HCoV-229E**	**1.3**	**2.8**	**16**	**Furin-like S2' cleavage site**
**G9G2X4**	**1**	**56**	**N**	**HCoV-HKU1**	**1.2**	**5.3**	**41**	**N-terminal RNA binding domain**
**Q01455**	**197**	**230**	**M**	**HCoV-OC43**	**1.2**	**3.5**	**147**	**Interaction with N protein region**
**Q6Q1R8**	**309**	**364**	**N**	**HCoV-NL63**	**1.1**	**9.5**	**132**	**C-terminus of dimerization domain**
**P0C6X2**	**4509**	**4564**	**pp1ab**	**HCoV-HKU1**	**1.1**	**3.6**	**2**	**RNA-directed RNA polymerase**
**Q0QJI4**	**757**	**812**	**S**	**HCoV-OC43**	**1.0**	**4.9**	**46**	**Receptor binding domain**
**Q5MQC5**	**29**	**84**	**ORF8**	**HCoV-HKU1**	**1.0**	**3.0**	**41**	**N-terminal RNA-binding domain**
**P0C6X2**	**1065**	**1120**	**pp1ab**	**HCoV-HKU1**	**0.9**	**2.8**	**12**	**Asp-rich region, Papain-like proteinase**
**P0C6X1**	**5377**	**5432**	**pp1ab**	**HCoV-229E**	**-2.2**	**2.4**	**9**	**RNA virus helicase**
**Q0ZJJ1**	**965**	**1020**	**pp1ab**	**HCoV-HKU1**	**-2.7**	**26.8**	**12**	**Asp-rich region, Papain-like proteinase**
**P0C6X4**	**897**	**952**	**pp1ab**	**HCoV-HKU1**	**-3.0**	**6.5**	**12**	**Asp-rich region, Papain-like proteinase**
**Q0ZJG7**	**925**	**980**	**pp1ab**	**HCoV-HKU1**	**-3.4**	**40.7**	**12**	**Asp-rich region, Papain-like proteinase**
**P0C6X4**	**925**	**980**	**pp1ab**	**HCoV-HKU1**	**-3.6**	**65.2**	**12**	**Asp-rich region, Papain-like proteinase**
**P0C6X3**	**925**	**980**	**pp1ab**	**HCoV-HKU1**	**-3.6**	**65.4**	**12**	**Asp-rich region, Papain-like proteinase**
**P0C6X4**	**953**	**1008**	**pp1ab**	**HCoV-HKU1**	**-4.0**	**81.9**	**12**	**Asp-rich region, Papain-like proteinase**
**P0C6X2**	**1037**	**1092**	**pp1ab**	**HCoV-HKU1**	**-4.0**	**66.6**	**12**	**Asp-rich region, Papain-like proteinase**
**P0C6X4**	**1009**	**1064**	**pp1ab**	**HCoV-HKU1**	**-4.1**	**89.8**	**12**	**Asp-rich region, Papain-like proteinase**
**Q0ZJJ1**	**1037**	**1092**	**pp1ab**	**HCoV-HKU1**	**-4.2**	**106.1**	**12**	**Asp-rich region, Papain-like proteinase**
**P0C6X3**	**1009**	**1064**	**pp1ab**	**HCoV-HKU1**	**-4.3**	**94.1**	**12**	**Asp-rich region, Papain-like proteinase**
**P0C6X2**	**951**	**1006**	**pp1ab**	**HCoV-HKU1**	**-4.3**	**114.6**	**12**	**Asp-rich region, Papain-like proteinase**
**P0C6X3**	**1037**	**1092**	**pp1ab**	**HCoV-HKU1**	**-4.4**	**105.0**	**12**	**Asp-rich region, Papain-like proteinase**
**P0C6X4**	**981**	**1036**	**pp1ab**	**HCoV-HKU1**	**-4.9**	**139.5**	**12**	**Asp-rich region, Papain-like proteinase**
**P0C6X2**	**925**	**980**	**pp1ab**	**HCoV-HKU1**	**-5.2**	**147.1**	**12**	**Asp-rich region, Papain-like proteinase**
**Q0ZJJ1**	**925**	**980**	**pp1ab**	**HCoV-HKU1**	**-5.3**	**153.3**	**12**	**Asp-rich region, Papain-like proteinase**
**P0C6X3**	**953**	**1008**	**pp1ab**	**HCoV-HKU1**	**-5.5**	**162.7**	**12**	**Asp-rich region, Papain-like proteinase**
**P0C6X3**	**951**	**1006**	**pp1ab**	**HCoV-HKU1**	**-5.9**	**183.5**	**12**	**Asp-rich region, Papain-like proteinase**
**Q0ZJJ1**	**1093**	**1148**	**pp1ab**	**HCoV-HKU1**	**-6.0**	**182.1**	**12**	**Asp-rich region, Papain-like proteinase**
**P0C6X2**	**949**	**1004**	**pp1ab**	**HCoV-HKU1**	**-6.2**	**192.8**	**12**	**Asp-rich region, Papain-like proteinase**
**Q0ZJJ1**	**953**	**1008**	**pp1ab**	**HCoV-HKU1**	**-6.2**	**190.9**	**12**	**Asp-rich region, Papain-like proteinase**
**P0C6X2**	**953**	**1008**	**pp1ab**	**HCoV-HKU1**	**-6.2**	**195.5**	**12**	**Asp-rich region, Papain-like proteinase**
**Q0ZJJ1**	**979**	**1034**	**pp1ab**	**HCoV-HKU1**	**-6.6**	**204.6**	**12**	**Asp-rich region, Papain-like proteinase**
**P0C6X4**	**4761**	**4816**	**pp1ab**	**HCoV-HKU1**	**-7.1**	**21.0**	**36**	**RNA-directed RNA polymerase**
**Q0ZJG7**	**4985**	**5040**	**pp1ab**	**HCoV-HKU1**	**-8.1**	**47.6**	**21**	**3C-like proteinase**
**P0C6X1**	**337**	**392**	**pp1ab**	**HCoV-229E**	**-9.8**	**128.6**	**219**	**Non-structural protein 2**

[A]Start and end position of enriched peptides relative to the amino acid sequences in UniProtKB. [B]Cluster annotation was adapted from UniProtKB entry descriptions and features. Only peptides with a log odds ratio (lod) ≥ ln(2) or ≤ ln(-2) and a *P* value < 0.005 (Fisher's exact test) are listed. Immunodominant peptides (i.e., peptides found to be significantly enriched in ≥1% of samples of the tested individuals (*N* = 1399)) are marked in bold font.

Supplemental Table 7. Demographic data and sample collection for COVID-19 patients

Patient no.	Age	Gender	Nationality	Time from onset of symptoms to sample collection
**1**	49 years	Female	Belgian	25 days, 55 days
**2**	68 years	Male	Pakistani	5 days
**3**	30 years	Male	Egyptian	12 days
**4**	49 years	Male	Indian	10 days
**5**	68 years	Male	Sudanese	9 days
**6**	56 years	Male	Indian	10 days
**7**	67 years	Male	Indian	12 days

Supplemental Table 8. Peptides enriched by PhIP-Seq in COVID-19 patients (*n* = 7)

UniProtKB entry	Start[A]	End[A]	Species	Protein symbol	Cluster no.	No. of samples enriched	Cluster annotation[B] / peptide location
**K9N5Q8**	841	896	MERS-CoV	S	16	2	Furin-like S2' cleavage site
**Q91A26**	869	924	Beta-CoV-1[C]	S	16	2	Furin-like S2' cleavage site
**Q5MQD0**	897	952	HCoV-HKU1	S	19	3	Furin-like S2' cleavage site
**Q14EB0**	897	952	HCoV-HKU2	S	19	3	Furin-like S2' cleavage site
**Q14EB0**	869	924	HCoV-HKU3	S	19	2	Furin-like S2' cleavage site
**A0A0U2V9P1**	85	140	MERS-CoV	S	19	2	Furin-like S2' cleavage site
**A0A023Y9K3**	813	868	MERS-CoV	S	19	3	Furin-like S2' cleavage site
**A0A023Y9K3**	841	896	MERS-CoV	S	19	3	Furin-like S2' cleavage site
**T2BBI8**	869	924	MERS-CoV	S	19	3	Furin-like S2' cleavage site
**P59594**	785	840	SARS-CoV	S	19	6	Furin-like S2' cleavage site
**Q91A26**	897	952	Beta-CoV-1[C]	S	19	4	Furin-like S2' cleavage site
**U5NJG5**	1177	1232	MERS-CoV	S	59	3	Heptad repeat 2
**U5NJG5**	1205	1260	MERS-CoV	S	59	3	Heptad repeat 2
**K9N5Q8**	1205	1260	MERS-CoV	S	59	2	Heptad repeat 2
**K9N5Q8**	337	392	MERS-CoV	S	71	2	Receptor binding region
**P59594**	1093	1148	SARS-CoV	S	91	4	Heptad repeat 2
**P59594**	1121	1176	SARS-CoV	S	91	6	Heptad repeat 2
**Q14EB0**	1149	1204	HCoV_HKU1	S	140	2	Upstream of heptad repeat 2
**Q91A26**	1205	1260	Beta-CoV-1[C]	S	181	3	Heptad repeat 2
**P59594**	533	588	SARS-CoV	S	245	2	C-terminal of receptor binding domain
**Q8BB23**	197	252	Beta-CoV-1[C]	N	15	2	SR-rich region
**P59595**	141	196	SARS-CoV	N	25	4	N-terminal RNA-binding domain
**P59595**	197	252	SARS-CoV	N	25	5	SR-rich region
**Q6Q1R8**	337	377	HCoV-NL63	N	132	2	C terminus
**P59595**	365	420	SARS-CoV	N	243	4	C terminus
**A0A075Q443**	169	224	MERS-CoV	pp1a	24	2	NA
**W6A0R5**	1121	1176	MERS-CoV	pp1a	57	2	Papain-like proteinase
**K9N7C7**	5489	5544	MERS-CoV	pp1ab	8	4	Helicase
**Q0ZJJ1**	953	1008	HCoV-HKU1	pp1ab	12	2	Asp-rich region, Papain-like proteinase
**P0C6X2**	953	1008	HCoV-HKU1	pp1ab	12	4	Asp-rich region, Papain-like proteinase
**P0C6X2**	949	1004	HCoV-HKU1	pp1ab	12	5	Asp-rich region, Papain-like proteinase
**P0C6X3**	951	1006	HCoV-HKU1	pp1ab	12	3	Asp-rich region, Papain-like proteinase

[A]Start and end position of enriched peptides relative to the amino acid sequences in UniProtKB. [B]Cluster annotation was adapted from UniProtKB entry descriptions and features. [C]Non-human CoV isolate. Only peptides that were significantly enriched in two or more patients are listed.
